# The Galectin-3-binding protein promotes angiogenesis in pancreatic cancer via simultaneous upregulation of VEGFA and direct HUVEC activation mediated by and VAMP5-STAT3

**DOI:** 10.1186/s12964-026-02801-7

**Published:** 2026-03-18

**Authors:** Ye Jin Lim, Dong Woo Son, Eun Ji Lee, Chae Won Yu, Yun Ok Oh, Min Jung Cha, Hyori Kim, Kyunggon Kim, Suhwan Chang

**Affiliations:** 1 Department of Physiology, BK21 Project, University of Ulsan Collage of Medicine, Seoul, South Korea; 2 Asan Institute for Life Sciences, Seoul, 05505 South Korea; 3 Department of Precision Medicine, University of Ulsan Collage of Medicine, Seoul, South Korea; 4 Asan Medical Center, Seoul, 05505 South Korea

**Keywords:** Galectin-3-binding protein, Angiogenesis, VAMP5, Pancreatic ductal adenocarcinoma, Antibody therapeutics

## Abstract

Pancreatic ductal adenocarcinoma (PDAC) is ranked by the lowest 5-year survival rate worldwide. Given the lack of early symptoms and diagnostic biomarkers, most patients present with either local or distant metastasis at diagnosis. We previously demonstrated that Galectin-3-binding protein (Gal-3BP) is overexpressed in PDAC cells and promotes metastasis through the EGFR pathway, using proteomic analysis of PDAC tumor interstitial fluid (TIF). In the present study, we show the Gal-3BP promotes angiogenesis in PDAC by vascular endothelial growth factor A (VEGFA) upregulation via STAT3 activation. Furthermore, we reveal the Gal-3BP directly promotes migration and tube formation of vascular endothelial cells through STAT3 phosphorylation, mediated not by a VEGF receptor but by VAMP5. To explore the clinical application of these findings, we developed a humanized, high-affinity Gal-3BP antibody to block the dual angiogenic function of Gal-3BP. The #132 clone of the Gal-3BP antibody significantly reduced blood vessel density in PDAC orthotopic tumors and attenuated PDAC metastasis in a lung metastasis model. Therefore, we demonstrate the antibody-mediated blockade of Gal-3BP attenuates the angiogenesis in PDAC and propose it as a novel therapeutic strategy.

**Supplementary Information:**

The online version contains supplementary material available at 10.1186/s12964-026-02801-7.

## Introduction

Pancreatic ductal adenocarcinoma (PDAC) accounts for approximately 90% of all pancreatic cancer cases [1, 2]. Most PDAC patients present with distant metastasis at diagnosis due to the lack of early symptoms and late diagnosis [3, 4]. Despite cytotoxic therapy (radiation and chemotherapy), PDAC patients exhibit the lowest 5-year survival among all cancer types worldwide [5, 6]. This low survival highlights the need for therapies that can more effectively inhibit PDAC metastasis [3]. The tumor microenvironment (TME) of PDAC is characterized by desmoplastic stroma comprising fibroblasts, blood vessels, and pancreatic stellate cells (PSCs) that surround cancer cells [7]. Immunotherapies for the treatment of solid tumors have recently showed significant advancements [8]. However, the efficacy of immunotherapy remains limited for PDAC patients due to specific challenges in the TME of PDAC [7, 9]. To overcome these challenges, biomarkers that can detect PDAC at an early stage and aid the development of new therapeutic strategies, including combination therapy, are critically needed [8].

Cancer secretome plays a key role as both a biomarker [10] and a therapeutic target of various cancer types [11, 12], including breast cancer [13], colorectal cancer [12], and pancreatic cancer [14]. It is crucial for cancer progression, angiogenesis, and metastasis [12, 15]. In PDAC, cancer secretome is associated with tumor viability, metastasis, and therapeutic resistance [11, 16]. Previous studies demonstrated that secreted proteins, which serve as potential biomarkers, are positively correlated with shorter survival in cancer patients [16]. Therefore, the cancer-derived secretome represents a promising biomarker for therapeutic targeting and disease stage monitoring in PDAC.

Galectin-3-binding protein (Gal-3BP) is a highly glycosylated protein that is abundantly secreted by cancer cells, being implicated in tumor progression and metastasis across multiple cancer types, including breast cancer [17], lung cancer [18], and pancreatic cancer [14, 19]. Several studies showed that elevated serum or tumor tissue Gal-3BP is associated with poor survival in patients with solid cancers [17]. Functionally, Gal-3BP and its related family members mediate cell-to-cell or cell-to-extracellular matrix (ECM) interactions [19]. Additionally, they promote tumor angiogenesis and metastasis through key signaling pathways, such as EGF/EGFR and PI3K/AKT signaling [14, 20]. Recent studies have suggested that Gal-3BP functions as a pro-angiogenic factor through the PI3K/AKT pathway, particularly in breast cancer [17] and endometrial cancer [21]. Nevertheless, its potential angiogenic role in PDAC remains unclear. Taken together, inhibiting Gal-3BP may offer potential to reduce metastasis and angiogenesis in PDAC.

Angiogenesis is the formation of new blood vessels from pre-existing ones [22, 23]. In cancer TME, cancer cells secrete pro-angiogenic factors such as vascular endothelial growth factor A (VEGFA), which promotes the migration and sprouting of vascular endothelial cells [24]. Anti-angiogenic therapies for PDAC have primarily targeted VEGF and its receptors [25]. Investigated anti-angiogenic agents in PDAC include monoclonal antibodies, such as ramucirumab (an anti-VEGFR2 antibody) [26] and bevacizumab (an anti-VEGF antibody) [6, 27]. However, no significant improvement in overall survival was observed in PDAC patients treated with anti-angiogenic drugs. Although PDAC is characterized by the low blood vessel density, angiogenesis remains crucial for tumor growth, proliferation, and metastasis [24, 28]. Recent studies have demonstrated that micro-vessel density (MVD) and VEGFA expression are positively correlated with tumor progression in PDAC [28, 29]. Therefore, anti-angiogenic therapies hold promise in inhibiting PDAC progression by blocking blood vessel growth.

In a previous study, we reported that Gal-3BP was highly expressed in PDAC-TIF compared to breast cancer, as determined by liquid chromatography-tandem mass spectrometry (LC-MS/MS). To control the function of Gal-3BP, we developed anti-Gal-3BP antibodies from an immunized chicken library using phage display technology, demonstrating their anti-metastatic effects in PDAC [14]. In this study, we found novel function of Gal-3BP in angiogenesis by controlling HUVEC. Additionally, we showed that engineered anti-Gal-3BP antibodies block PDAC metastasis and angiogenesis significantly, suggesting the antibody mediated blockade of Gal-3BP as a promising therapeutic strategy for PDAC metastasis.

## Results

### Anti-Galectin-3-binding protein antibodies attenuate PDAC metastasis

**Fig. 1:**
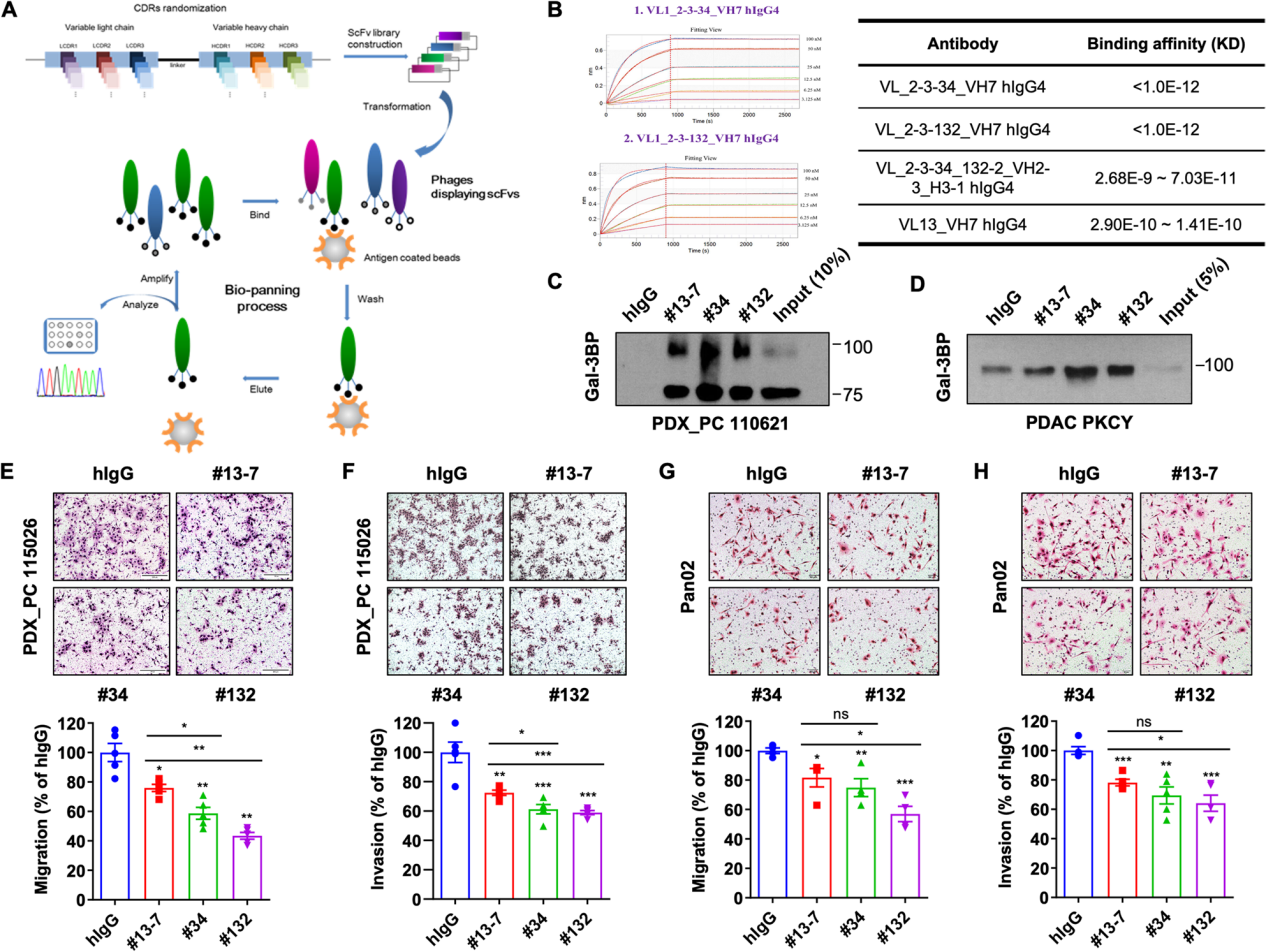
Affinity matured, anti-Gal-3BP antibodies show superior in vitro efficacy for blocking PDAC cell migration and invasion. **A**. Schematic diagram of antibody affinity maturation of the humanized anti-Gal-3BP antibody. The #13 − 7 clone was used as a template. Two high-affinity clones, #34 and #132, were tested in further study. **B**. Affinity of the engineered #34 and #132 antibody clones measured by Octet (on left) and their value (Table on right). The affinity of parental clone #13 − 7 is shown at the bottom of the table. **C**–**D**. Validation of Gal-3BP binding of engineered anti-Gal-3BP antibodies (#13 − 7, #34, and #132), shown by immunoprecipitation in PDX_PC 110,621 (**C**) or PDAC PKCY (**D**). **E**–**F**. Migration and invasion of PDX_PC 115,026 treated with engineered anti-Gal-3BP antibodies (#13 − 7, #34, and #132) or hIgG (human IgG). Upper panel shows representative images, and lower graphs present analysis data. Scale bar: 200 μm. **G**–**H**. Migration and invasion of Pan02 treated with engineered anti-Gal-3BP antibodies (#13 − 7, #34, and #132) or hIgG. Upper panel shows representative images, and lower graphs present analysis data. Scale bar: 50 μm.* *p* < 0.05; ** *p* < 0.01; *** *p* < 0.001; ns (not significant)

In the previous study, we generated anti-Gal-3BP antibodies by immunizing chickens and evaluated their metastasis inhibitory effects [14]. We developed affinity-matured anti-Gal-3BP antibody clones (#34 and #132) from a humanized version of the chicken #84 antibody (#13 − 7) using CDR randomization and bio-panning (Fig. 1A). These antibodies validated the enhanced binding affinity, as confirmed by Octet analysis (Fig. 1B) and immunoprecipitation (Fig. 1C and D). We evaluated the migration and invasion of PDAC cells treated with the engineered antibodies to assess their improved inhibition of PDAC metastasis. Both humanized and affinity-matured anti-Gal-3BP antibody clones significantly attenuated migration and invasion in human PDAC cells, including PDX_PC 115,026 (Fig. 1E and F), PDX_PC 110,621 (Supplementary Fig. 1A), and BxPC-3 (Supplementary Fig. 1B), as well as in mouse PDAC cells, Pan02 (Fig. 1G and H), and PDAC PKCY (Supplementary Fig. 1C). The affinity-matured antibodies showed stronger inhibitory effects on PDAC cell migration and invasion compared to the humanized antibody (#13 − 7). Interestingly, clone #132 demonstrated a stronger inhibitory effect on migration and invasion of PDAC cells compared to #13 − 7 and #34, even at a low concentration of 0.5 µg/ml (Supplementary Fig. 1D). However, those antibodies did not reduce the proliferation of PDAC cells (Supplementary Fig. 1E) and a co-culture between PDAC cells and PSCs (Supplementary Fig. 1F).

### Gal-3BP positively regulates VEGFA expression in PDAC

**Fig. 2:**
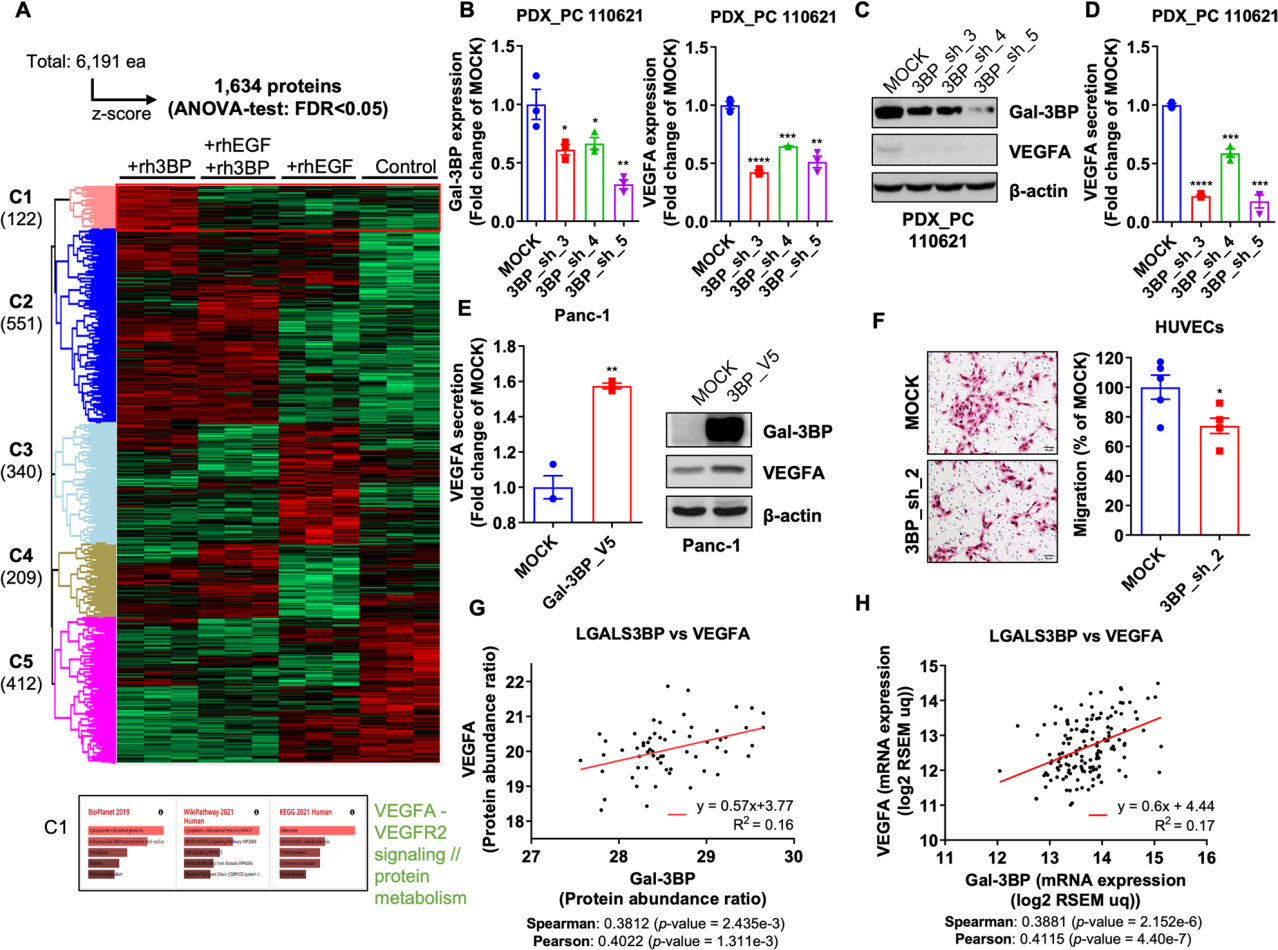
Gal-3BP positively regulates VEGFA production in PDAC. **A**. Clustered heatmap of the protein expression profile for PDX_PC 110,621 treated with rhGal-3BP, rhEGF, or rhGal-3BP+rhEGF combination. Red indicates upregulated protein levels, whereas green shows downregulated cases. The bottom panel indicates the VEGFA-VEGFR2 pathway enriched in C1 cluster, as shown by WikiPathway 2021 analysis. **B** and **C**. mRNA (**B**) and protein expression (**C**) of VEGFA in PDX_PC 110,621 after Gal-3BP knockdown by shRNA. **D**. VEGFA secretion measured by ELISA in PDX_PC 110,621 after Gal-3BP knockdown. **E**. The secretion of VEGFA measured in Panc-1 cells after Gal-3BP overexpression. **F**. Transwell migration of HUVEC co-cultured with control PDX_PC 110,621 or with Gal-3BP knockdown. The graph on right shows relative migration rate. Scale bar: 50 μm. **G**–**H**. Expression correlation analysis between Gal-3BP and VEGFA for mRNA (**G**) or protein (**H**), using cBioportal database.* *p* < 0.05; ** *p* < 0.01; *** *p* < 0.001; **** *p* < 0.0001; ns (not significant)

In the previous study, we reported that extracellular Gal-3BP promotes PDAC metastasis via the EGF/EGFR pathway [14]. We performed proteomic analyses following treatment with rhGal-3BP (recombinant human Gal-3BP) in PDAC cells to investigate the molecular mechanisms associated with extracellular Gal-3BP on PDAC. When Gal-3BP was added to the culture medium of PDAC cells, the VEGFA/VEGFR2 signaling pathway was activated (Fig. 2A). Likewise, the VEGFA/VEGFR2 signaling pathway was downregulated in PDAC cells treated with anti-Gal-3BP chicken antibody (#84) (Supplementary Fig. 2A). The VEGFA/VEGFR2 pathway promotes angiogenesis in cancer [30, 31]. Then, we evaluated VEGFA expression in PDAC cells following *LGALS3BP* knockdown to investigate the relationship between Gal-3BP and VEGFA. Both mRNA and protein VEGFA levels were significantly downregulated following shRNA-mediated *LGALS3BP* knockdown in PDX_PC 110,621 cells (Fig. 2B and C) and PDX_PC 115,026 (Supplementary Fig. 2B and 2 C). Similarly, secreted VEGFA levels were reduced following *LGALS3BP* knockdown in PDX_PC 110,621 cells (Fig. 2D). In contrast, VEGFA secretion and protein levels were upregulated in LGALS3BP-overexpressing Panc-1 (Fig. 1E) and PDX_PC 17,884 (Supplementary Fig. 2D). Afterward, PDX_PC 110,621 cells were indirectly co-cultured with HUVECs to mimic the interaction between blood vessels and PDAC cells. *LGALS3BP* knockdown in PDAC cells significantly reduced the migration ability of HUVECs compared to the control (Fig. 2F). Figure 2G and H demonstrate a positive correlation of Gal-3BP with VEGFA mRNA and protein expression in PDAC tissue. Thus, Gal-3BP promotes angiogenesis by positively regulating VEGFA expression in PDAC.

### Extracellular Gal-3BP promotes VEGFA expression in PDAC by regulating STAT3 phosphorylation

**Fig. 3:**
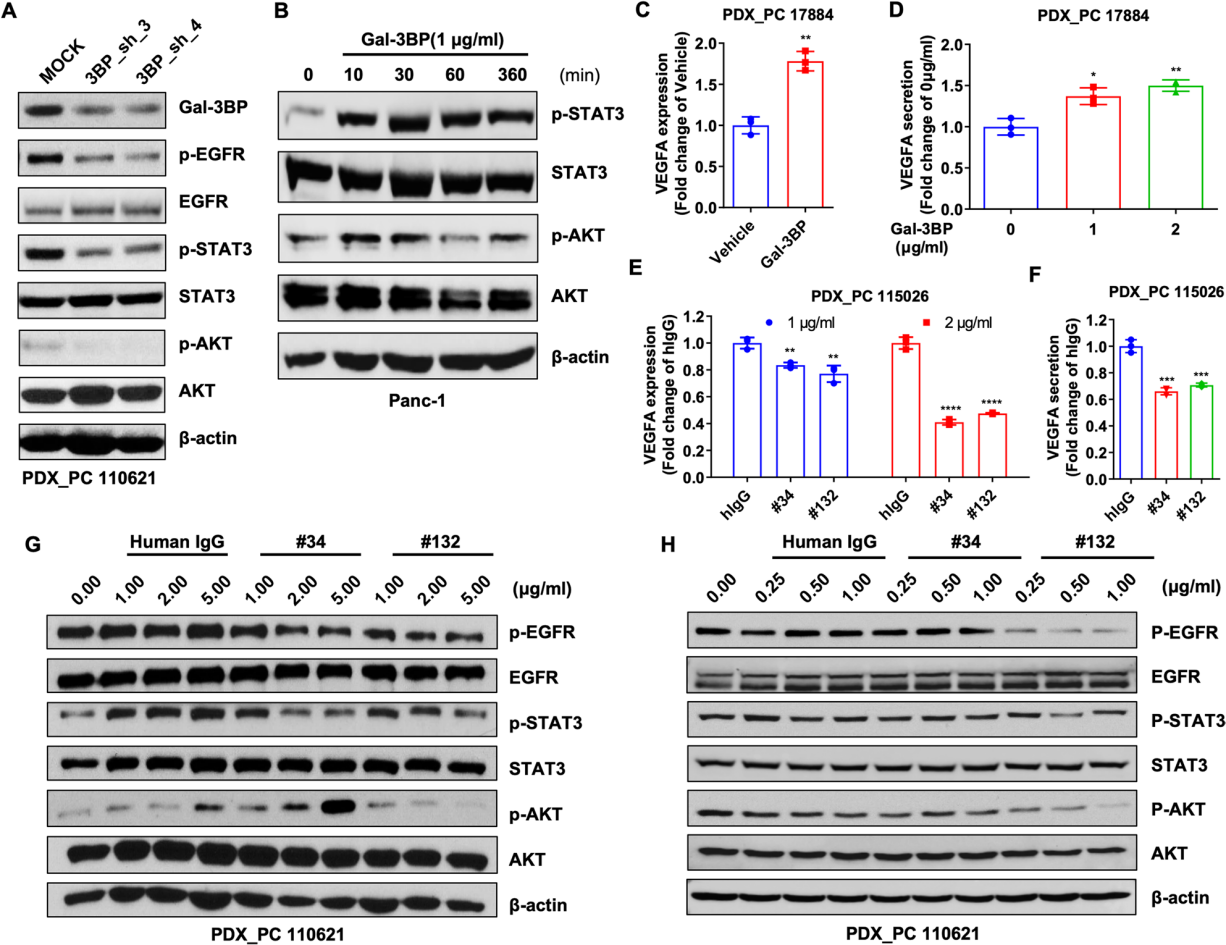
STAT3/AKT activation mediates VEGFA production induced by Gal-3BP and is reversed by the engineered anti-Gal-3BP antibodies. **A**. Activation of EGFR, STAT3, and AKT in PDX_PC 110,621 with Gal-3BP knockdown, as analyzed by Western blot using phosphoantibodies. **B**. Activation profile of AKT and STAT3 determined by Western blot in Panc-1 stimulated with rhGal-3BP (1 µg/ml). b-actin was used as a loading control. **C** and **D**. mRNA (**C**) and secreted VEGFA levels (C and **D**) in rhGal-3BP-stimulated PDX_PC 17,884 cells. **E** and **F**. Engineered Gal-3BP antibody reduces VEGFA mRNA expression (**E**) and secretion (**F**) in PDAC cell PDX_PC 17,884. **G** and **H**. The #34 or #132 Ab inactivate EGFR and STAT3 in PDX_PC 110,621. The cells were treated with 1–5 µg/ml (**G**) or 0.25–1 µg/ml (**H**) and analyzed by Western blot. * *p* < 0.05; ** *p* < 0.01; *** *p* < 0.001; **** *p* < 0.0001; ns (not significant)

A previous study showed that AKT and STAT3 are regulators of VEGFA expression through EGFR in cancer [32, 33]. We analyzed phosphorylation levels in PDAC cells following shRNA-mediated LGALS3BP knockdown to validate whether Gal-3BP induces VEGFA expression through STAT3 and AKT phosphorylation in PDAC. EGFR, AKT, and STAT3 phosphorylation was downregulated in LGALS3BP-knockdown PDAC cells compared to the control (Fig. 3A). Then, we examined EGFR, AKT, and STAT3 phosphorylation in rhGal-3BP-stimulated Panc-1 cells to explore the mechanism by which extracellular Gal-3BP regulates VEGFA expression in cancer cells. Gal-3BP stimulation markedly increased the phosphorylation of AKT and STAT3 (Fig. 3B). Furthermore, we validated that extracellular Gal-3BP promotes VEGFA expression through autocrine effects in PDAC cells. We then measured VEGFA secretion in rhGal-3BP-treated PDX_PC 17,884 cells, which exhibit low endogenous Gal-3BP expression, to investigate Gal-3BP-mediated VEGFA regulation in PDAC. Both VEGFA mRNA levels and secretion were significantly increased upon extracellular Gal-3BP stimulation in PDX_PC 17,884 (Fig. 3C and D). Conversely, VEGFA mRNA levels were downregulated in anti-Gal-3BP antibodies–treated PDX_PC 115,026 (Fig. 3E) and PDX_PC 110,621 (Supplementary Fig. 2E). Secreted VEGFA was also reduced in PDX_PC 115,026 treated with anti-Gal-3BP antibodies (#34 and #132) (Fig. 2F). Additionally, we evaluated VEGFA expression and AKT and STAT3 phosphorylation following antibody treatment in PDAC cells to assess whether anti-Gal-3BP antibodies reduce VEGFA expression by inhibiting AKT and STAT3 phosphorylation. Anti-Gal-3BP antibodies downregulated EGFR, STAT3, and AKT phosphorylation in PDX_PC 110,621 (Fig. 3G and H) and BxPC-3 (Supplementary Fig. 2F). Interestingly, antibody treatment inhibited pEGFR levels at concentrations above 1 µg/ml for both #34 and #132, while phosphorylated STAT3 levels decreased at concentrations above 0.25 µg/ml. Notably, #132 reduced AKT phosphorylation while #34 did not show such effect (Fig. 3G and H). Therefore, we concluded extracellular Gal-3BP positively regulates VEGFA expression through STAT3 phosphorylation via autocrine effects in PDAC. This data is further validated by ChIP assay for the VEGFA promoter (Supplementary Fig. 3A) or VEGFA promoter luciferase assay (Supplementary Fig. 3B), activated by Gal-3BP treatment. In addition, we examined the Gal-3BP-STAT3 can affect the expression of EMT markers in PDAC cells, as we have observed the Gal-3BP antibody inhibits PDAC migration and invasion (Fig. 1E and H). The data in Supplementary Figure S4 shows increased N-Cadherin, Vimentin and TWIST upon Gal-3BP treatment, that is reversed by STAT3 inhibitor S31-201 (Supplementary Figure S4A, S4B).

### Gal-3BP induces migration and tube formation of vascular endothelial cells via p-STAT3

**Fig. 4:**
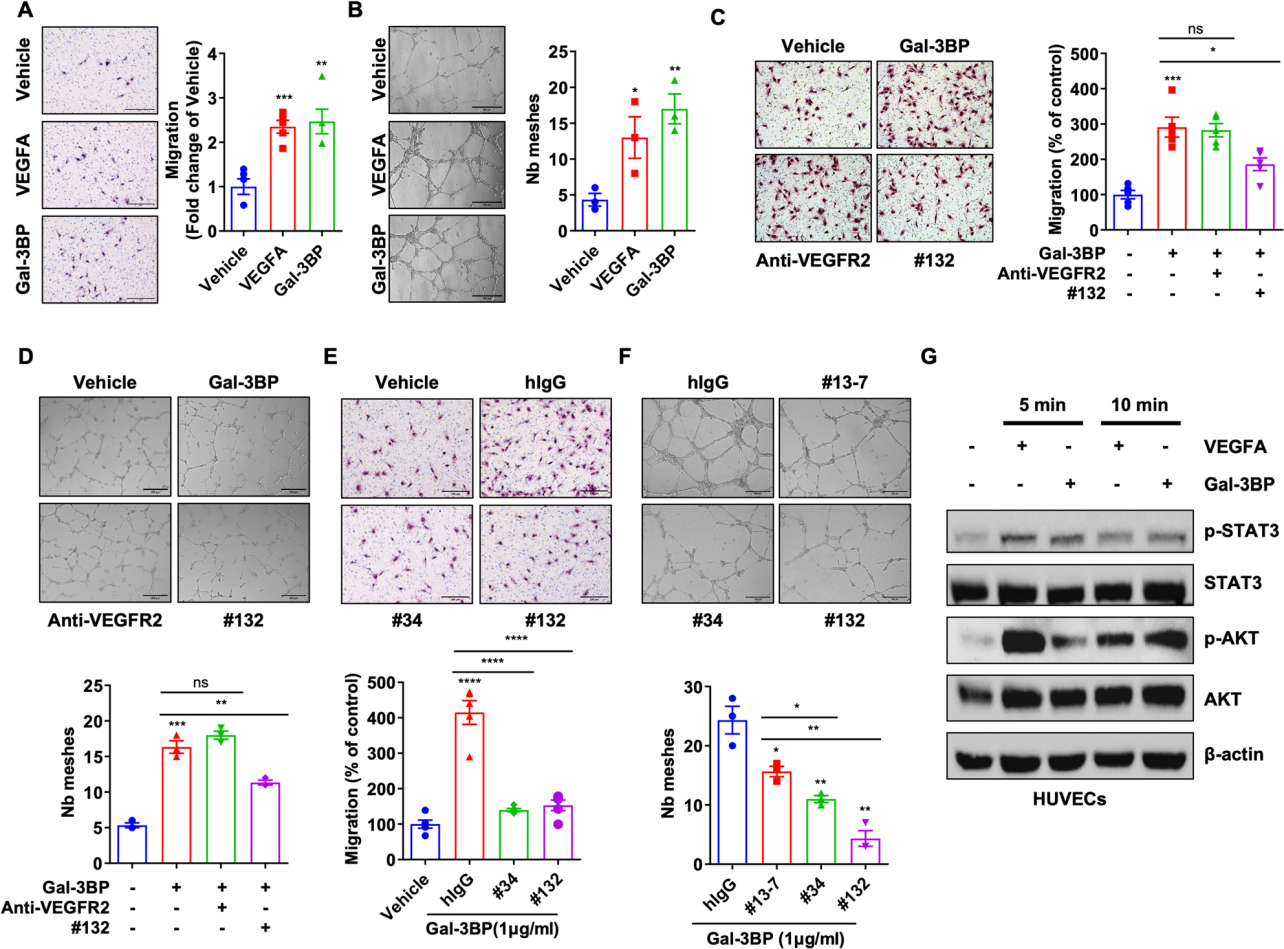
Gal-3BP activates STAT3/AKT and promotes migration and tube formation of HUVEC cells. **A**. Increased migration of HUVEC cells treated with VEGFA (20 ng/ml) or Gal-3BP (1 µg/ml). Scale bar: 200 μm. Graph on right shows the relative migratory activity. **B**. Increased tube formation of HUVEC cells treated with VEGFA (20 ng/ml) or Gal-3BP (1 µg/ml). Scale bar: 200 μm. Graph on right shows number of meshes counted from the images. **C** and **D**. #132, but not anti-VEGFR2 antibody (ramucirumab), can reverse the increased migration (**C**) or tube formation (**D**) of HUVEC cells, which was induced by Gal-3BP treatment. Upper panel shows representative images, and lower graphs present analysis data. Graphs at bottom show the relative migratory activity (**C**) or the number of meshes (**D**). Scale bar: 200 μm. **E**. Migration assay of Gal-3BP-stimulated HUVEC cells in combination with anti-Gal-3BP antibodies (#34 and #132) or hIgG. Upper panels show representative images, and lower graphs present analysis data. Bottom graphs show relative migratory activity. Scale bar: 200 μm. **F**. Tube formation assay of HUVEC cells in PDX_PC 115,026-conditioned media in the presence of anti-Gal-3BP antibodies (#13 − 7, #34 and #132) or hIgG. Scale bar: 200 μm. **G**. Western blot analysis of p-STAT3, STAT3, p-AKT, AKT, and β-actin in VEGFA (20 ng/ml) or Gal-3BP (1 µg/ml)-stimulated HUVEC cells. * *p* < 0.05; ** *p* < 0.01; *** *p* < 0.001; **** *p* < 0.0001; ns (not significant)

Several studies identified extracellular Gal-3BP as a pro-angiogenic factor in various cancer types [17, 21]. Accordingly, we conducted migration, tube formation, and proliferation assays using HUVEC cells to investigate whether Gal-3BP plays a similar role in promoting angiogenesis in PDAC. Gal 3BP significantly enhanced HUVEC migration comparable to VEGFA (Fig. 4A). Similarly, Gal-3BP increased the number of meshes in the tube formation assay, indicating its pro-angiogenic effect (Fig. 4B). In contrast, Gal-3BP supplementation did not promote HUVEC proliferation in the proliferation assay, which assessed the combined effects of Gal-3BP, galectin-3, and VEGFA (Supplementary Fig. 5). Thus, the pro-angiogenic activity of Gal-3BP is primarily attributed to its effects on migration and tube formation rather than HUVEC proliferation. We also examined a neutralizing antibody for VEGFA, Bevacizumab, and measured its effect on Gal-3BP induced HUVEC migration or tube formation. The results in Supplementary Fig. 6 show Bevacizumab significantly reduces Gal-3BP mediated HUVEC migration or tube formation, confirming the role of VAGFA in the Gal-3BP induced HUVEC activation.

To further investigate whether extracellular Gal-3BP activates migration and tube formation via VEGFR2, we used Ramucirumab, an anti-VEGFR2 monoclonal antibody commonly employed in anti-angiogenic therapy. We first checked the efficacy of Ramucirumab (Supplementary Fig. 7A, 7B), that significantly inhibits HUVEC migration or tube formation. We also observed the Ramucirumab restored activated p-ERK induced by VEGFA (Supplementary Fig. 7C). While ramucirumab effectively inhibited HUVEC migration and tube formation by VEGFA, it did not block rhGal-3BP-induced migration and tube formation (Fig. 4C and D). However, the data in Supplementary Fig. 8A shows the activation of VEGFR2 by Gal-3BP treatment that is reversed by either #132 Ab or VEGFR2 TKI inhibitor SU-5408. Consistently, downstream p-ERK also showed similar regulation pattern (Supplementary Fig. 8B). Even though SU-5408 inhibits not only VEGFR2 but also FGFR1 (IC50 30nM), these data suggest the possibility of VEGFR2 involvement in the migration and tube formation of HUVEC cells. This is further supported by additional data in Supplementary Fig. 8C and 8D, showing SU-5408 reverse Gal-3BP induced HUVEC migration similarly to #132 Ab. Another clone #34 also showed similar effect in reducing HUVEC migration (Fig. 4E) and tube formation (Fig. 4F). These data demonstrates that Gal-3BP promotes HUVEC migration and tube formation and it is partially blocked by either anti-Gal-3BP antibody or VEGFR2 TKI SU-5408. We next questioned if STAT3 is involved in this regulation similar to PDAC cells. We treated rhGal-3BP with HUVECs and the result shows Gal-3BP stimulation upregulated AKT and STAT3 phosphorylation (Fig. 4G). These data demonstrate Gal-3BP secreted from PDAC cell promotes angiogenesis through AKT and STAT3 phosphorylation in HUVEC.

## VAMP5 mediates the proangiogenic function of secreted Gal-3BP on vascular endothelial cells

**Fig. 5:**
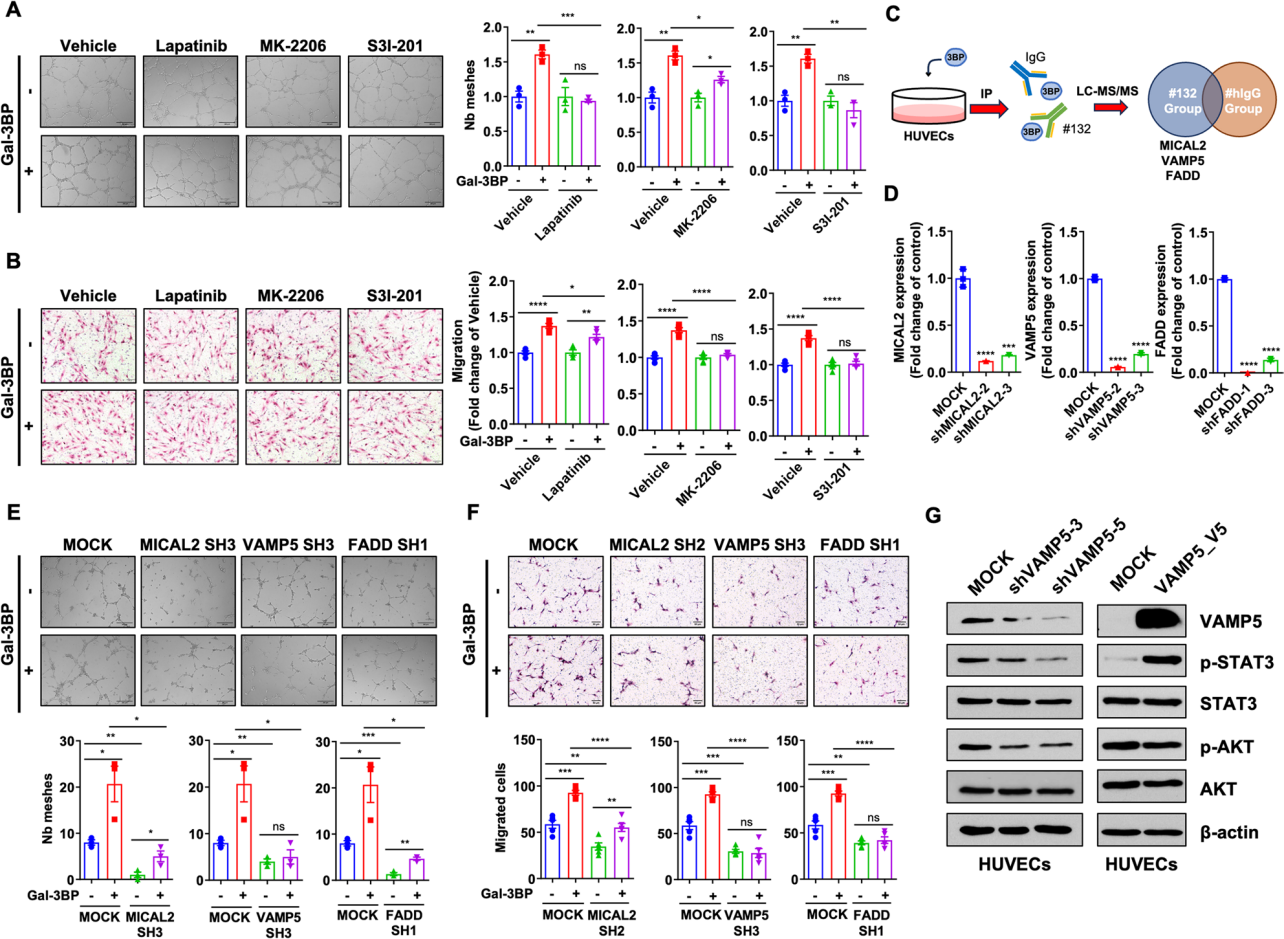
VAMP5 is a mediator for the pro-angiogenic effect of Gal-3BP in HUVEC cells. **A**. Tube formation assay of HUVEC cells treated with Vehicle (DMSO), lapatinib (1 µM, left graph), MK-2206 (100 nM, middle graph), and S3I-201 (30 µM, right graph) in the presence of rhGal-3BP (1 µg/ml). Left panels show representative images, and graphs on right present data analysis. Scale bar: 200 μm. **B**. HUVEC migration assay with Vehicle, lapatinib (1 µM, left graph), MK-2206 (100 nM, middle graph), S3I-201 (30 µM, right graph) treatment in the presence of rhGal-3BP (1 µg/ml). Left panels show representative images, and graphs on right present data analysis. Scale bar: 50 μm. **C**. Schematic diagram for IP-LC-MS/MS analysis to identify receptor protein of secreted Gal-3BP on HUVEC cells. Engineered anti-Gal-3BP antibody #132 was used, and top 3 candidates were listed. **D**. MICAL2, VAMP5, and FADD mRNA levels in HUVEC cells with shRNA-mediated knockdown. **E** and **F**. Tube formation and migration assay of HUVEC cells depleted with MICAL2, VAMP5, and FADD after treatment with rhGal-3BP (1 µg/ml, marked as Gal-3BP[+]). Upper panel shows representative images, and lower graphs present data analysis. Scale bar: 200 μm (tube formation assay) and 50 μm (migration assay). **G**. Western blot for VAMP5, p-STAT3, STAT3, p-AKT, AKT, and β-actin in shRNA-targeted *VAMP5* knockdown of HUVEC cells (left) and *VAMP5*-overexpressing HUVEC cells (right). * *p* < 0.05; ** *p* < 0.01; *** *p* < 0.001; **** *p* < 0.0001; ns (not significant)

To validate the extracellular Gal-3BP positively regulates AKT and STAT3 phosphorylation in HUVEC, we utilized MK-2206 (an AKT inhibitor), S3I-201 (a STAT3 inhibitor), and lapatinib (an EGFR and ERBB2 inhibitor) and asked whether STAT3 and AKT activation play critical roles in HUVEC migration and tube formation. The concentration of each inhibitor was determined based on its inhibitory effects on the target phosphorylation (Supplementary Fig. 9A–C). We selected a maximum dose that does not affect HUVEC proliferation (Supplementary Fig. 9D–E). As a result, we found S3I-201 significantly reduced the number of meshes in the rhGal-3BP-induced HUVEC tube formation (Fig. 5A). In contrast, MK-2206 exhibited a comparatively weaker effect on reducing tube formation. Furthermore, both MK-2206 and S3I-201 significantly reduced rhGal-3BP-induced HUVEC migration (Fig. 5B). Altogether, S3I-201 (a STAT3 inhibitor) exhibited a consistent effect in the Gal-3BP-induced HUVEC tube formation. Hence, we conclude STAT3 activation plays a dominant role in Gal-3BP-mediated angiogenesis.

To identify the receptor of Gal-3BP on HUVEC, we performed immunoprecipitation using #132 Ab, followed by proteomic analysis using LC-MS/MS. Our analysis revealed MICAL2, FADD, and VAMP5 as potential candidates interacting with Gal-3BP on vascular endothelial cells (Fig. 5C). Consistent with our IP-Mass results, VAMP5 was identified as interacting partner of Gal-3BP in human cell lines [34]. This interaction is also validated in HEK293 cells by co-immunoprecipitation (Supplementary Fig. 10A) and colocalization of Gal-3BP and VAMP5, by immunocytochemistry staining (Supplementary Fig. 10B), without upregulation in its RNA level (Supplementary Fig. 10C). We then performed shRNA-mediated knockdown of *MICAL2*, *FADD*, and *VAMP5* to examine the role of these candidates in HUVEC migration and tube formation. The knockdown was validated by the reduced mRNA expression of the target genes (Fig. 5D). Interestingly, *MICAL2*, *FADD*, and *VAMP5* knockdown all significantly reduced the tube formation of HUVECs (Fig. 5E). Notably, VAMP5 depletion abolished the rescue of tube formation by recombinant Gal-3BP treatment, whereas it was partially rescued in MICAL2- and FADD-depleted cells (Fig. 5E). We observed a similar effect of VAMP5 depletion in the HUVEC migration assay (Fig. 5F). Thus, we concluded VAMP5 is a critical protein for the Gal-3BP-mediated migration and tube formation in HUVEC cells. VAMP5 plays a critical role in vesicle transport [35], but its function in cancer has not been fully understood. As we observed the secreted Gal-3BP activates STAT3/AKT in HUVEC, we next questioned if the VAMP5 is involved in this pathway. The result in Fig. 5G indicate the VAMP5-depleted HUVECs have reduced STAT3 and AKT phosphorylation. Conversely, VAMP5 overexpression led to increased STAT3, but not AKT, phosphorylation (Fig. 5G, right panels). Therefore, extracellular Gal-3BP promotes migration and tube formation in HUVECs via VAMP5-STAT3 activation.

### Anti-Gal-3BP antibodies reduce proliferation and angiogenesis in a PDAC orthotopic model

**Fig. 6:**
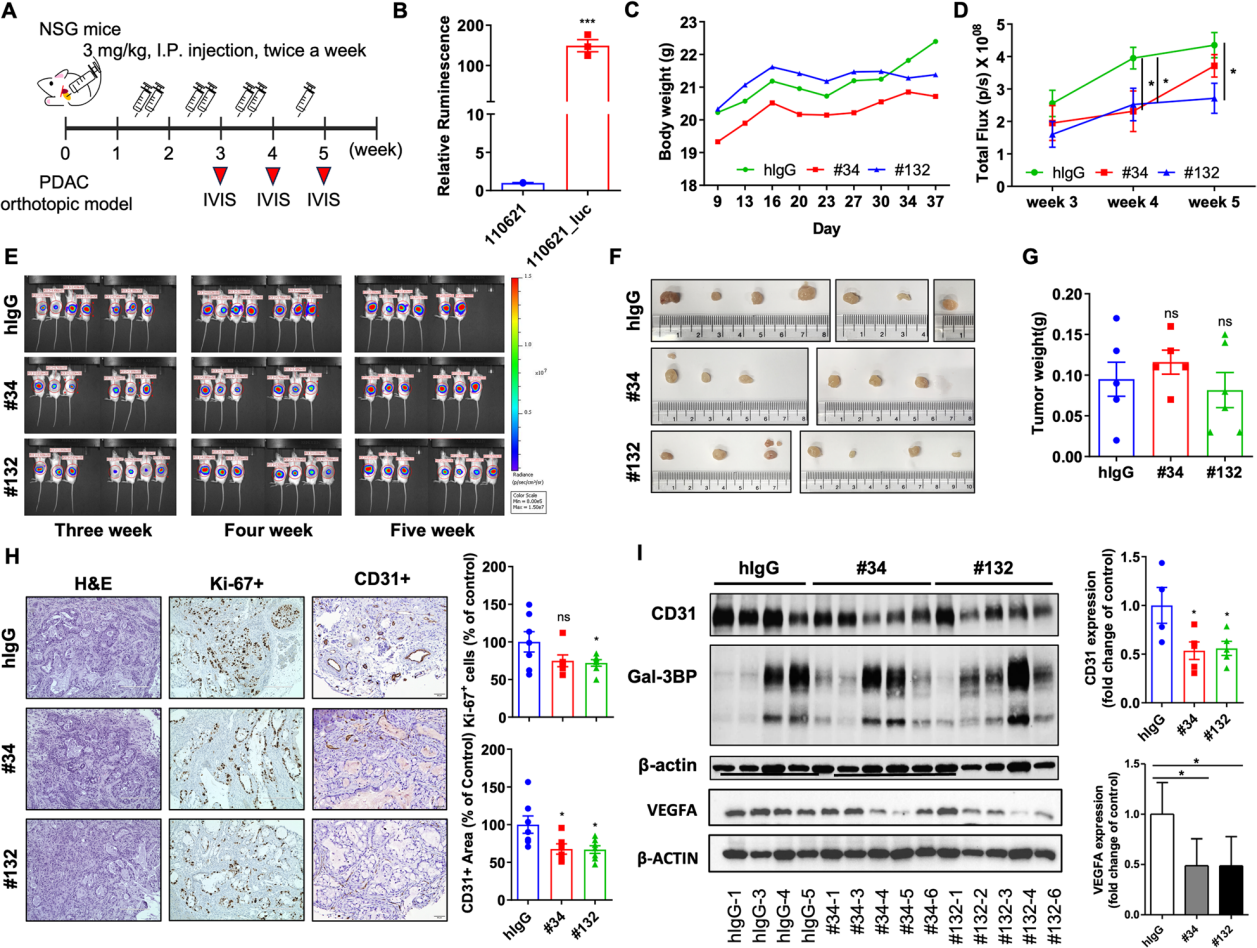
Engineered anti-Gal-3BP antibodies attenuate the in vivo angiogenesis of PDAC. **A**. Schematic representation of the PDAC orthotopic model and treatment procedure of the Gal-3BP antibodies (#34 and #132) along with live imaging schedule using IVIS. **B**. Luciferase assay of the PDX_PC 110,621 cells with luciferase stable expression (110621_luc). **C**. Body weight measurements for each group during the antibody treatment (*n* = 20). **D** and **E**. A graph of a ROI value (**D**) indicating tumor progression between week 3 (start of antibody treatment) and week 5 (end of antibody treatment) along with representative IVIS images for each treatment group (**E**) from week 3 to week 5 (*n* = 20). **F**. Images of dissected PDAC orthotopic tumors from each antibody treatment group. **G**. Graph showing the average tumor weight for each antibody treatment group. **H**. Immunohistochemistry analysis of Ki-67 and CD31 in orthotopic tumors. Representative images are shown on the left panels, and their relative signal was presented in the graphs on right. Scale bar: 200 μm (H&E and Ki-67 staining) and 50 μm (CD31 staining) (*n* = 20). **I**. Western blot analysis of CD31, Gal 3BP, and VEGFA expression levels in harvested tumors from each group. Graphs on right presents densitometry results for VEGFA or CD31 normalized by b-actin. * *p* < 0.05; *** *p* < 0.001; ns (not significant)

PDAC is characterized by fibroblastic microenvironments with limited anti-tumor immunity, presenting significant therapeutic challenges due to its desmoplastic nature [36, 37]. Validating in vivo efficacy is critical to assess the therapeutic potential of Gal-3BP antibodies. To determine dose for the in vivo experiment, we performed dose-dependent migration inhibition assay (Supplementary Fig. 11) and tube formation assay (Supplementary Fig. 12) in HUVEC, ranging from 0.2 to 5µg/ml. In addition, a PDAC orthotopic model was developed to evaluate the effect of affinity-matured antibodies on PDAC (Fig. 6A). The PDX_PC 110621_luc cells, which had been transduced with a luciferase fusion-retrovirus, were used to monitor tumor progression via IVIS Spectrum imaging. PDX_PC 110621_luc cells exhibited significantly higher luciferase expression compared to control cells (Fig. 6B). Orthotopic pancreatic tumors were generated by injecting 5 × 10^5^ of PDX_PC 110621_luc cells into the pancreas of NSG mice. One week after the injection, the mice received intraperitoneal injections of either affinity-matured antibodies (#34 and #132) or human IgG (3 mg/kg) twice a week. Tumor growth was monitored once a week by IVIS imaging. There was no significant difference in body weight between control and Gal-3BP antibody-treated groups (Fig. 6C). Bioluminescence, measured as the total flux in the region of interest (ROI) in an IVIS image (Fig. 6D and E), revealed a significant decrease in cancer cell bioluminescence in the #132 treated group at 4 and 5 weeks after the cancer cell injection. In contrast, the #34-treated group exhibited reduced bioluminescence at 4 weeks; however, this effect was not statistically significant at 5 weeks. At 5 weeks after the injection, mice were euthanized, and their tumors were collected (Fig. 6F). Tumor weight in the #132 treated group was lower than that in the control group, although without a statistically significant difference. The #34-treated group showed no significant reduction in tumor weight (Fig. 6G). Replicating the PDAC microenvironment in vitro is challenging. Therefore, we analyzed Ki-67 (a marker of cell proliferation) and CD31 (a blood vessel marker) expression in orthotopic tumors. Immunohistochemistry revealed that Ki-67 expression was significantly lower in the #132-treated group compared to the control group, while the #34-treated group showed decreased Ki-67 expression (Fig. 6H, upper graph). CD31 staining results demonstrated a reduction in the CD31-positive area in both anti-Gal-3BP antibody-treated groups (Fig. 6H, lower graph). Western blot analysis confirmed these findings, showing a significant decrease in CD31 expression in both #34 and #132-treated groups (Fig. 6I, lower graph). VEGFA expression was also reduced in the anti-Gal-3BP antibody-treated group in orthotopic tumors (Fig. 6I, upper graph). In summary, treatment with anti-Gal-3BP antibodies reduced tumor cell proliferation and angiogenesis in PDAC orthotopic tumors.

### Anti-Gal-3BP antibodies attenuate metastasis of PDAC cells in mouse model

**Fig. 7:**
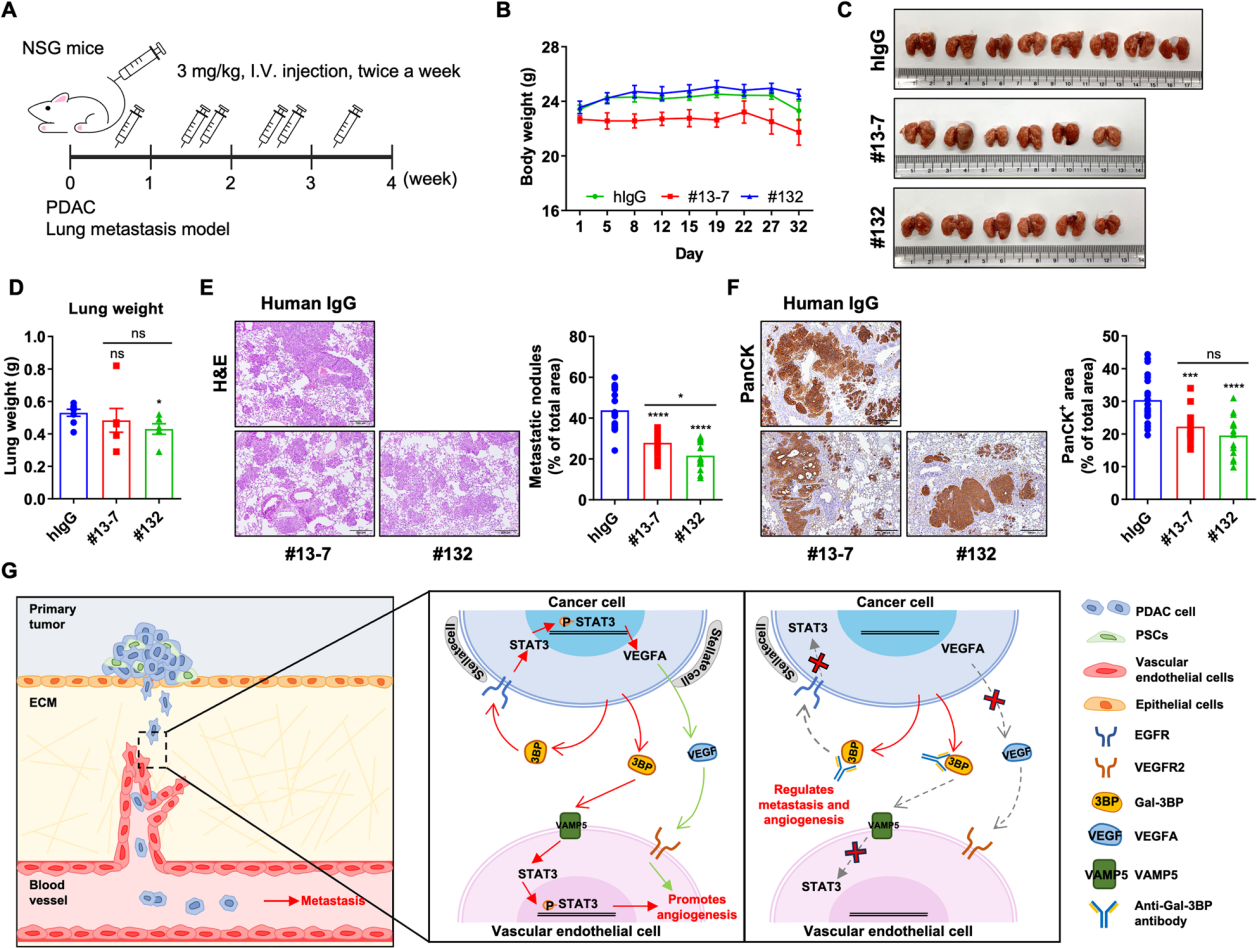
Engineered anti-Gal-3BP antibodies abrogate PDAC metastasis in vivo. **A**. Schematic diagram of the PDAC lung metastasis experiment using a tail-vein injection of BxPC3 PDAC cells along with the treatment schedule of #13 − 7 (*n* = 6), #132 (*n* = 6), and control IgG (*n* = 8). **B**. Body weight of each treatment group during the experiment period (*n* = 20). **C** and **D**. Images of lungs (**C**) and their weight (**D**) at the endpoint from each treatment group. **E** and **F**. Metastasis index (**E**) or PanCK-positive area (**F**) measured by immunohistochemistry of the lungs from each treatment group. Representative images are shown on left panels, and the graphs on right show data analysis. Scale bar: 200 μm. **G**. Graphical summary of the study, indicating the role of extracellular Gal-3BP in promoting angiogenesis by upregulating VEGFA secretion from cancer cells (autocrine effect) or directly interacting with VAMP5 on HUVEC (paracrine effect). * *p* < 0.05; *** *p* < 0.001; **** *p* < 0.0001; ns (not significant)

Affinity-matured anti-Gal-3BP antibodies attenuate PDAC migration and invasion more effectively than before affinity maturation (Fig. 1). A lung metastasis model was established using BxPC-3 cells to investigate the inhibitory effect of anti-Gal-3BP antibody on metastasis. BxPC-3 cells (1 × 10^6^) were intravenously injected into the tail vein of the NSG mice to induce lung metastasis. Then, anti-Gal-3BP antibodies (#13 − 7 and #132) or control IgG were intravenously injected into the tail vein of mice twice a week (Fig. 7A). No significant difference in body weight was observed among the mice during antibody treatment (Fig. 7B). After 6 antibody injections, lung weight was reduced in the #132-treated group compared to the control IgG-treated group (Fig. 7C and D). Hematoxylin and eosin (H&E) staining of lung tissue showed that the metastatic nodule area in the anti-Gal-3BP antibodies (#13 − 7 and #132)-treated group was reduced compared to the control IgG-treated group (Fig. 7E). Furthermore, metastatic nodules showed a significant decrease in the #132 antibody-treated group compared to the #13 − 7 antibody-treated group. panCK expression, a cancer cell marker, was also suppressed in the anti-Gal-3BP antibody-treatment groups (#13 − 7 and #132) (Fig. 7F). Hence, anti-Gal-3BP antibody represents a promising strategy to inhibit metastasis and angiogenesis in the PDAC preclinical model (Fig. 7G).

## Discussion

Gal-3BP, as a protein secreted by cancer cells, promotes metastasis in several cancers, including PDAC [14, 19, 38]. Blocking extracellular Gal-3BP is considered critical for inhibiting such process. Research on anti-Gal-3BP antibodies has expanded to various cancers, including lung cancer [38], breast cancer [39], and pancreatic cancer [40]. Capone et al. and Dufrusine et al. developed anti-Gal-3BP antibody-drug conjugates and evaluated their anti-cancer effects in glioblastoma [41], adenoid cystic carcinoma [42], and neuroblastoma [43]. Despite these advancements, functional studies on antibody-mediated Gal-3BP blockade in PDAC remain limited. Previously, we developed two anti-Gal-3BP antibodies by immunizing chicken and demonstrated their anti-metastatic effects in PDAC [14]. To advance these antibodies for therapeutic applications, we conducted humanization and affinity maturation for enhancing antigen binding, improving sensitivity and interaction stability under physiological conditions [44]. Humanized anti-Gal-3BP antibodies (clone #84) underwent these steps, resulting in two anti-Gal-3BP antibodies used in this study (#34 and #132).

These antibody clones effectively could bind to extracellular Gal-3BP and significantly reduce the migration and invasion of PDAC cells compared to the humanized antibody clone (#13 − 7). However, anti-Gal-3BP treatment did not inhibit PDAC proliferation in vivo (Fig. 6G and H). Of note, our proteomic analysis demonstrated that extracellular Gal-3BP upregulates VEGFA/VEGFR2 signaling, which is involved in cancer angiogenesis, including PDAC [30, 45]. Hence, we investigated the positive correlation between Gal-3BP and VEGFA expression in PDAC. Extracellular Gal-3BP promotes VEGFA expression through the EGFR/STAT3 pathway in PDAC. Additionally, we found that VEGFA level was downregulated in PDAC following treatment with anti-Gal-3BP antibodies. The angiogenic role of Gal-3BP was supported by its ability to enhance vascular endothelial cell migration and tube formation via STAT3 phosphorylation. These results are consistent with previous studies reporting that Gal-3BP promotes VEGFA expression in breast and endometrial cancers [17, 21]. Furthermore, our proteomic analysis identified VAMP5 as a potential mediator of Gal-3BP-induced STAT3 phosphorylation in vascular endothelial cells, although direct interaction between Gal-3BP and VAMP5 remains to be confirmed. Nevertheless, VAMP5 was associated with vascular endothelial cell migration and tube formation, indicating its potential involvement in Gal-3BP-mediated angiogenesis.

To explore a possible EGFR activation by Gal-3BP in HUVEC, we tested conditioned media of PDAC in combination with CTX (Cetuximab; EGFR blocker) on HUVEC and examined EGFR/p-EGFR, STAT3/p-STAT3 by western blot. The results in Supplementary Fig. 13 shows undetectable p-EGFR level, with unaltered EGFR expression (detected in longer exposure, see graph on B). Therefore, we think the EGFR signaling is not responsible for the Gal-3BP induced HUVEC activation. In contrast, when we checked p-STAT3/STAT3, we observed it is increased by PDAC conditioned media but not blocked by CTX (EGFR blocker). These data suggest that the activation of STAT3 in HUVEC is not mediated by EGFR.

We generated the PDAC orthotopic model and PDAC lung metastasis model treated with anti-Gal-3BP antibodies to assess the inhibitory efficacy of anti-Gal-3BP antibodies in PDAC an in vivo model. The tail vein injection model is acute and has limitation compared with spontaneous model, but considering the role of anti-Gal-3BP in HUVEC tube formation and angiogenesis in tumor (Fig. 6H), the effect Gal-3BP antibody on lung colonization implies its anti-angiogenic effect in vivo. Indeed, the treatment of anti-Gal-3BP antibodies reduced CD31 expression in orthotopic tumors, demonstrating their inhibitory effect on tumor angiogenesis. While In vitro experiments did not confirm a reduction in cancer cell proliferation by anti-Gal-3BP antibodies, anti-Gal-3BP antibodies (#34 and #132 clones) at a dose of 3 mg/kg significantly decreased Ki-67 expression in PDAC orthotopic tumors. Thus, these antibodies may inhibit tumor cell proliferation under specific conditions. In the lung metastasis model, affinity-matured anti-Gal-3BP antibody (#132) inhibited PDAC metastasis more effectively compared to #13 − 7 antibody. These results underscore the necessity of dose optimization for maximizing the therapeutic efficacy of anti-Gal-3BP antibodies for primary tumor. Furthermore, given the critical role of angiogenesis in PDAC progression, targeting Gal-3BP could provide a novel approach for anti-angiogenic and anti-metastatic therapy.

PDAC is characterized by a desmoplastic and heterogeneous TME, which poses significant challenges to effective treatment. Despite advances in PDAC therapies, such as gemcitabine, FOLFIRINOX (a regimen that includes folinic acid, fluorouracil, irinotecan hydrochloride, and oxaliplatin), or the combination of nab-paclitaxel with gemcitabine, PDAC remains one of the deadliest cancers, with 5-year survival below 10% [46, 47]. Our study suggests that while anti-Gal-3BP antibodies effectively reduce PDAC metastasis and angiogenesis, they may not sufficiently suppress primary tumor growth as a monotherapy. Thus, future studies should explore combining anti-Gal-3BP antibodies with existing chemotherapeutic or immunotherapeutic therapies to enhance their therapeutic potential.

Overall, our study presents an important step in demonstrating the therapeutic potential of anti-Gal-3BP antibody in PDAC treatment. However, several limitations must be resolved in future work. First, while we demonstrated the involvement of STAT3 phosphorylation in Gal-3BP-mediated angiogenesis, the underlying molecular mechanism remains to be fully elucidated. Second, the lack of direct evidence for the interaction between Gal-3BP and VAMP5 requires further studies to validate its role as a mediator of Gal-3BP signaling in vascular endothelial cells as well as raise a possibility for other receptor involvement in this process. Finally, more dose-dependent study with toxicity evaluation as well as exploring combination therapies with anti-Gal-3BP antibody will be essential for translating it into clinical applications.

## Materials and methods

### Ethical guidelines and human sample acquisition

Human PDAC specimens were obtained and identified with permission from the Institutional Review Board of the Asan Medical Center (S2013-0744-0009). The Institutional Animal Care and Use Committee approved the protocol for animal experiments (IACUC, Project Number: 2023-02-017). All mice were maintained in a specific pathogen-free facility at the Laboratory of Animal Research at Asan Institute for Life Sciences (Seoul, South Korea).

### Cell culture

Patient-derived xenograft pancreatic cancer cells (PDX_PC), such as 115,026 and 110,621, were cultured in RPMI 1640 medium (SH30027.01, Cytiva) supplemented with 5% fetal bovine serum (FBS) (SH30919.03, Cytiva), 1% penicillin/streptomycin (SV30010, HyClone), 20 ng/mL of epidermal growth factor (EGF) (AF-100-15-500, Peprotech), 4 µg/mL of hydrocortisone (H0888-1G, Sigma-Aldrich), and 4 µg/mL of transferrin (T3705-1G, Sigma-Aldrich). HEK 293 cells, Platinum-A cells, PDAC PKCY, and Panc-1 were cultured in Dulbecco’s Modified Eagle Medium (DMEM) (SH30243.01, Cytiva) with 10% FBS and 1% penicillin/streptomycin. BxPC-3 was cultured in the RPMI 1640 medium supplemented with 10% FBS and 1% penicillin/streptomycin. Pan02 was cultured in the RPMI 1640 medium supplemented with 15% FBS and 1% penicillin/streptomycin. Human PSCs were cultured on Stellate Cell Medium (5301, ScienCell). Human umbilical vein endothelial cells (HUVECs) were purchased from Lonza (C2519A) and maintained in Endothelial Cell Basal Medium-2 (EBM-2) (CC-3156, Lonza) supplemented with EGM-2 SingleQuots Supplements (CC-4176, Lonza). Human pancreatic duct epithelial cell line (HPDE) was cultured in Keratinocyte-SFM (10724-011, Gibco) with supplements for keratinocyte-SFM (37000-015, Gibco). All cells were cultured at 37 °C in an atmosphere containing 5% CO_2_.

### LC-MS/MS analysis

LC-MS/MS was used to analyze the protein, which binds with extracellular Gal-3BP on vascular endothelial cells. method of LC-MS/MS is described in previously study [14].

### shRNA-mediated knockdown or overexpression of the target gene

Plasmids containing shRNA against human LGALS3BP were purchased from Sigma Aldrich (#2 TRCN0000372778; #3 TRCN0000029417; #4 TRCN0000372838; #5 TRCN0000029414). HEK293 cells were co-transfected with a lentiviral expression vector and lentiviral packaging vectors (PMD2.G and PAX2) using Lipofectamine 3000 (L3000075, Invitrogen) to generate lentiviruses. The supernatants of co-transfected HEK293 cells were collected three times at 12-h intervals. For the transduction of PDX_PC 115,026, PDX_PC 110,621, PDX_PC 17,884, and Panc-1 cells, lentiviruses were added to the culture medium of the target cells along with hexadimethrine bromide (H9268, Sigma-Aldrich) at a concentration of 4–8 µg/ml for 2 days. Finally, puromycin (P8833, Sigma-Aldrich) or blasticidin (15205, Sigma-Aldrich) was added to the cultured media as a selection pressure to select cells with stable knockdown or overexpression. For the transduction of HUVECs, supernatants containing lentivirus from co-transfected HEK293 cells were collected using RPMI 1640 medium supplemented with 10% FBS. The lentivirus supernatant was mixed with EGM-2 medium at a 1:1 ratio in the presence of hexadimethrine bromide and incubated for 2 days.

### Tube formation assay

Matrigel was added to a 48-well culture plate (20048, SPL) and incubated for 1 h at 37 °C and 5% CO_2_. Suspended HUVECs were prepared in the EBM-2 medium. Cell suspensions, containing 4ⅹ10^4^ cells per well, were added to the Matrigel-coated 48-well plate with recombinant proteins or inhibitors. Lapatinib (S2111, Selleckchem), MK-2206 (S1078, Selleckchem), and S3I-201 (S1155, Selleckchem) were pre-treated on HUVECs 1 h before recombinant human Gal-3BP (rhGal-3BP) stimulation. rhGal-3BP and recombinant human VEGF (rhVEGF) were obtained from R&D systems (rhGal-3BP; AF2226-GAB, rhVEGF;293-VE). After 7-h incubation, three images were captured for analysis. Tube formation was analyzed using the Angiogenesis analyzer in Image J.

### Migration, invasion, and proliferation assay

The migration and invasion abilities of PDAC cells and HUVECs were assayed by using transwell chambers (3422, Corning) and Matrigel (354234, Corning). For the invasion assay, 200 µg/ml Matrigel was loaded on a coated transwell at 37 °C in an atmosphere containing 5% CO_2_. Subsequently, the cell suspension was added to each of the upper wells containing serum-free medium or culture media supplemented with serum or recombinant proteins serving as a chemoattractant in the lower wells. The chambers were incubated at 37 °C for either 24–48 h. For the co-culture migration assay, PDX_PC 110,621 cells were seeded in a 24-well culture plate. After 24 h, PDX_PC 110,621 cells were treated with 1 µg/ml mitomycin C (M4287, Sigma-Aldrich) in a complete medium for 4 h to inhibit cell division. HUVECs suspensions were added to the transwell inserts and co-cultured for 48 h. After incubation, the transwell chambers were stained with hematoxylin (S2-5, YD Diagnostics) and eosin (318906, Sigma-Aldrich). The number of migrated and invaded cells was quantified using the cell counter program in Image J.

For the proliferation assay, PDAC cells or PDAC cell and PSC co-culture were seeded in a 96-well culture plate and incubated at 37℃ for 24, 48, and 72 h. Following incubation, 1/10 volume of WST-8 reagent from the Quanti-MAX WST-8 Cell Viability Assay Kit (QM1000, BIOMAX) was added after each time point and incubated for 2 h at 37 °C. The absorbance was measured using a microplate reader at 450 nm.

### Immunoprecipitation

The proteins of PDX_PC 110,621 cells were harvested using EB buffer (20 mM HEPES, 0.1 M NaCl, 1 mM EDTA, 0.1% Triton X-100, 1 mM Dithiothreitol, and a protease inhibitor cocktail). For extracellular Gal-3BP in mouse PDAC cells, the cultured media were collected from a 150-mm culture dish containing serum-free medium. An aliquot of 500 µg of protein was placed in a 1.5-ml microcentrifuge tube, followed by adding 1 µg of each human IgG (NPA544Hu01, Cloud-Clone Corp.) and Gal-3BP-specific antibodies (13 − 7, 34, and 132). Samples were incubated overnight at 4℃ on a rotator. The following day, 20 µl of washed protein GSepharose 4 Fastflow (17061801, Cytiva) with phosphate-buffered saline (PBS) was added to each sample and incubated on the rotator at 9 rpm for 2 h at 4℃. After incubation, the samples were centrifuged at 2500 rpm for 5 min at 4℃, and the supernatant was removed. Subsequently, 700 µl of EB buffer was added, and the samples were incubated for 5 min on the rotator at 4℃. After 5 min, the samples were centrifuged again at 2500 rpm for 2 min at 4℃. This washing step was repeated 5 times. After the final wash, the samples were spun down, and the supernatant was thoroughly removed. Finally, 30 µl of 1X sample buffer was added to each sample, and the samples were boiled for 10 min at 98℃. Then, the supernatant was transferred to a new 1.5-ml microcentrifuge tube, and protein samples were loaded onto an SDS-Page gel.

### Protein quantification and western blot

The cells were lysed in a RIPA buffer with protease and phosphatase inhibitors. Cell lysates were centrifuged at 14,000 rpm for 15 min, and the supernatants were collected. Protein concentration was quantified using the Protein Quantification Kit (BCA0500, BIOMAX). The immunoblotting was performed with anti-Gal-3BP antibody (AF2226, R&D systems), anti-VEGFA antibody (ab46154, Abcam), anti-p-EGFR antibody (3777, Cell Signaling Technology), anti-EGFR antibody (4267, Cell Signaling Technology), anti-p-AKT antibody (9271, Cell Signaling Technology), anti-AKT antibody (9272, Cell Signaling Technology), anti-p-STAT3 antibody (9145, Cell Signaling Technology), anti-STAT3 antibody (4904, Cell Signaling Technology), anti-β-actin antibody (Santa Cruz Biotechnology), anti-VAMP5 antibody (ab216044, Abcam), and anti-CD31 antibody (77699, Cell Signaling Technology). For chemiluminescence detection on the membrane, the membranes were reacted with WestGlowTM PICO PLUS (BWP0200, BIOMAX) and WestGlowTM FEMTO (BWF0200, BIOMAX). The relative densities of bands were analyzed with ImageJ software.

### RNA extraction and quantitative real-time PCR

Total RNA extraction was performed using Tri-RNA Reagent (FATRR 001, FAVORGEN) following the manufacturer’s protocol. Total RNA was converted into cDNA using the PrimeScript RT reagent Kit (RR037A, TaKaRa) according to the manufacturer’s protocol. Gene expression was quantified using the EzAmp Real-Time qPCR 2X Master Mix (EBT-1801, ELPIS-BIOTECH) on the CFX Connect Real-Time System (BIO RAD). Relative expression levels were calculated using the 2-ΔΔCt method, with data presented as fold changes compared to the control. Table 1 summarizes the primer sequences for RT-qPCR.

**Table 1:** Primer sequences for RT-qPCR

Primer for RT-qPCR	
Primer	Sequence
LGALS3BP_RT_F2	5’- CCAATGAAACCAGGAGCACC − 3’
LGALS3BP_RT_R2	5’- CCAGGTTGGCAGTCAGGAT − 3’
hVEGFA_F	5’- TCTTCAAGCCATCCTGTGTG − 3’
hVEGFA_R	5’- TGCATTCACATTTGTTGTGC − 3’
MICAL2_RT_F	5’- CCATCACCGCCAACTTCATA − 3’
MICAL2_RT_R	5’- AGTGGGTGCAGTCCTTGTAG − 3’
VAMP5_RT_F	5’- GAATAGAGTTGGAGCGGTGC − 3’
VAMP5_RT_R	5’- GGCCAGGTTCTGTGTAGTCT − 3’
FADD_RT_F	5’- CTGGGGAAGAAGACCTGTGT − 3’
FADD_RT_R	5’- TGCGTTCTCCTTCTCTGTGT − 3’
hGAPDH_qPCR_F	5’- CCCATGTTCGTCATGGGTGT − 3’
hGAPDH_qPCR_R	5’- CCCATGTTCGTCATGGGTGT − 3’

### Immunocytochemistry

HUVEC cells were seeded in coverslip in 24well plate, incubated 24 h, and then washed with PBS. And fixation with 2% PFA in PBS at RT for 10 min incubation. Cells were incubated for 2 h with anti VAMP5 conjugated FITC (Assay lite,32854-05141T) and Gal-3BP antibody. After PBS washed and then incubated for 1 h with Alexa647-conjugated anti-mouse IgG antibodies (Invitrogen, A-21235). Fixed cells on coverslips were stained with DAPI for 1 min at room temperature, and the coverslips were then mounted onto glass slides. Fluorescence microscope images were obtained using a Carl Zeiss 710 Spectral Confocal Microscope.

### Dual luciferase VEGF reporter assay

Dual-luciferase reporter assays were performed in PANC-1 cells transfected with either the pGL4.28 empty vector or the pGL4-VEGF promoter reporter plasmid, together with an SV40-Renilla luciferase plasmid as an internal control. After 48 h of recovery, cells were treated with recombinant human Gal-3BP for 24 h. Luciferase activities were then measured using the Dual-Luciferase Reporter Assay System (Promega) according to the manufacturer’s instructions. Briefly, cells (2 × 10^5) were lysed in 100 µL of Passive Lysis Buffer, and 20 µL of cell lysate was transferred to a white 96-well plate. Firefly luciferase activity was measured following the addition of 100 µL of Luciferase Assay Reagent II, after which Renilla luciferase activity was measured following the addition of 100 µL of Stop & Glo^®^ reagent.

#### Chromatin immunoprecipitation (ChIP)–qPCR assay

Chromatin immunoprecipitation (ChIP) assays were performed to evaluate the binding of phosphorylated STAT3 (p-STAT3) to the VEGF promoter in PANC-1 cells. PANC-1 cells were treated with control (CON), recombinant Gal-3BP (1 µg/mL), STAT3 inhibitor (#132 Ab), or a combination of #132 Ab and Gal-3BP. For combination treatment, cells were pretreated with #132 Ab for 10 min, followed by Gal-3BP stimulation (1 µg/mL) for 1 h. Following treatment, cells were crosslinked with formaldehyde, and chromatin was extracted and sheared to an average length of 200–500 bp. Immunoprecipitation was performed using an anti–p-STAT3 antibody, with normal rabbit IgG used as a negative control. After reversal of crosslinking and DNA purification, ChIP-enriched DNA was analyzed by quantitative PCR (qPCR) using primers specific for the VEGF promoter region. ChIP enrichment was quantified by qPCR using VEGF promoter–specific primers (VEGF Fwd-1: 5′-CTGGCCTGCAGACATCAAAGTGAG-3′; VEGF Rev-1: 5′-CTTCCCGTTCTCAGCTCCACAAAC-3′) and is presented as relative enrichment normalized to rabbit IgG.

### Enzyme-linked immunosorbent assay

The PDAC cells were incubated in RPMI 1640 supplemented with 5% FBS for 24 h. Cell supernatants were collected after passing through a 0.45-µm syringe filter (SFCA03045, SOOMBIO). Secreted VEGF was detected using the Human VEGF ELISA Kit (DVE00, R&D Systems). The assay was performed according to the manufacturer’s protocol. The absorbance was measured using a microplate reader at 450 nm.

### Generation of luciferase-tagged pancreatic cancer cells

PDX_PC 110,621 cells expressing luciferase were generated using a retrovirus system. The pMXs-IRES-Puro (RTV-014) containing luciferase was transfected into the Platinum-A Retrovirus Packaging Cell line (RV-102, Cell Biolabs) using Lipofectamine 3000. For transduction, target cells were incubated in a retrovirus medium supplemented with Hexadimethrine bromide. The transduced cells were incubated in complete medium containing puromycin to select cells expressing luciferase. Subsequently, we performed the Dual-Luciferase^®^ Reporter (DLR™) Assay (E1960, Promega) to validate luciferase expression.

### Antibody treatment of the pancreatic cancer orthotopic model

Ten-week-old male NSG mice were obtained from JA Bio Laboratories (Suwon, South Korea). For the pancreatic cancer orthotopic model, PDX_PC 110,621 cells with stable luciferase expression (designated as PDX_PC 110621_luc) were utilized. The PDX_PC 110621_luc cells were resuspended at a density of 5 × 10^5^ cells/50 µl of Matrigel and injected into the mouse pancreas using a 26G 1-ml syringe. Antibody treatment was administered in the following three groups: control IgG-treated (*n* = 7), #34 antibody-clone-treated (*n* = 6), and #132 antibody-clone-treated (*n* = 7). The antibodies were intraperitoneally injected twice a week (3 mg/kg in PBS, pH 7.0) starting at 1-week post-surgery. Bioluminescence was analyzed using the IVIS spectrum imaging system. The mice were euthanized after the seventh injection, and the tumors were analyzed.

### Antibody treatment of the pancreatic cancer lung metastasis model

Ten-week-old male NSG mice were acquired from JA Bio Laboratories (Suwon, South Korea). BxPC-3 cells (1 × 10^6^ cells) were resuspended and injected into the mouse tail vein using a 26G 1-ml syringe. Antibodies were administered in the following three groups: human IgG (*n* = 8), #13 − 7 (*n* = 6), and #132 (*n* = 6). Antibodies were intravenously injected twice a week (3 mg/kg in PBS, pH 7.0) starting at 5 days after the cancer cell injection. Six antibody injections were performed. The mice were sacrificed 5 weeks after the cancer cell injection, and their lungs were analyzed.

### Immunohistochemistry

Fresh tissues were fixed in a 10% formalin solution (HT501320-9, Sigma-Aldrich) for 3 days and embedded in paraffin. Each paraffin block was sectioned to a thickness of 4 μm. The paraffin sections were deparaffinized using xylene (25165S0480, JUNSEI) and rehydrated with graded alcohol (100%–70%) (1.00983.2511, Sigma-Aldrich). The rehydrated sections were incubated in 3% hydrogen peroxide (H1067, Biosesang) containing 10% methanol (Dukan/MDEO-21018) for 20 min to block endogenous peroxidase activity. Antigen retrieval was performed in a citrate buffer (pH 7.0) (CBB999, TmBio). After antigen retrieval, the slides were incubated in a 1% normal horse serum for 1 h to block non-specific binding. Then, the slides were incubated with anti-CD31 antibody (77699, Cell Signaling Technology), anti-Ki67 antibody (Ab46667, Abcam), or anti-panCK antibody (Ab86734, Abcam) in 1% normal horse serum at 4℃ overnight. The samples were washed with PBS containing 0.1% Tween20 for 5 min to remove unbound antibodies. For DAB staining, we utilized the ABC-HRP Kit, peroxidase (PK-6200, VECTASTAIN^®^), and the Liquid DAB + Substrate Chromogen System (K3468, Dako) according to the manufacturer’s protocol. After DAB staining, we performed nuclear staining with hematoxylin on the tissue sections for 30 s. The stained tissue sections were dehydrated using graded alcohol (70%–100%) and xylene. Finally, the slides were mounted with Permount (SP15-100, Fisher Scientific), and 3 images were captured per slide at 20× magnification.

### Statistical analysis

An unpaired two-tailed Student’s t-test was performed by ANOVA for statistical analysis. All data are presented as mean + standard deviation (SD) or standard error of the mean (SEM). Correlations between variables were assessed using both Pearson’s correlation and Spearman’s correlation. *P*-values < 0.05 were considered statistically significant. *P*-values < 0.05, *p* < 0.01, *p* < 0.001, and *p* < 0.0001 are designated as *, **, ***, and ****, respectively.

## Supplementary Information

Supplementary Material 1.

## Data Availability

No datasets were generated or analysed during the current study.
