# Supplementary material for: The Galectin-3-binding protein promotes angiogenesis in pancreatic cancer via simultaneous upregulation of VEGFA and direct HUVEC activation mediated by and VAMP5-STAT3
Source: Cell Commun Signal. 2026 Mar 18;24:254. doi: 10.1186/s12964-026-02801-7 (PMC13112773; doi:10.1186/s12964-026-02801-7)
Supplement: Supplementary file 1 — Supplementary Material 1. [file 12964_2026_2801_MOESM1_ESM.pdf]

Figure S1

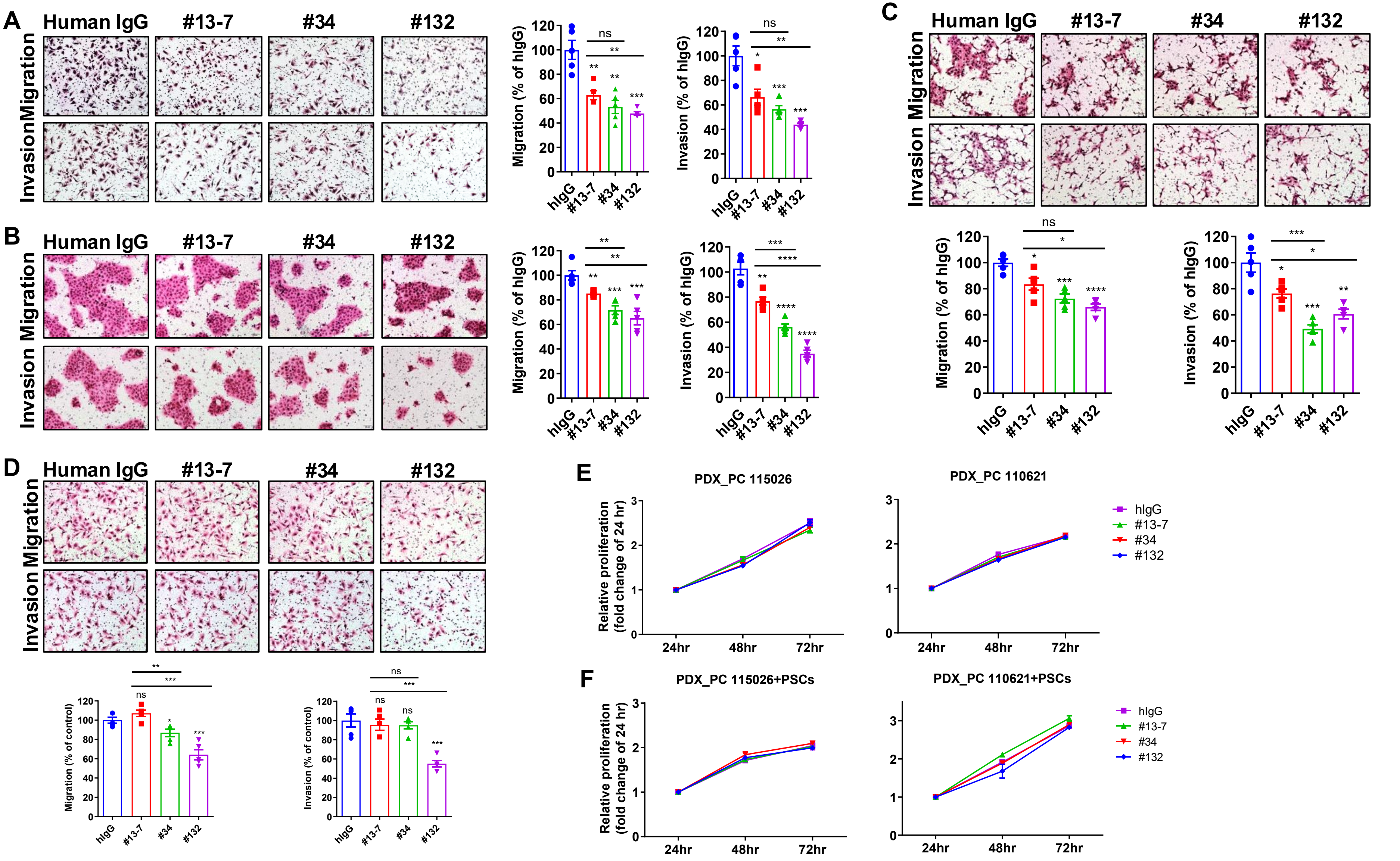

Supplementary Figure 1. Anti-Gal-3BP antibodies attenuate migration and invasion of PDAC cells

A - C. Migration and invasion assay of PDX_PC 110621 (A), BxPC-3 (B), and PDAC PKCY (C) with anti-Gal-3BP antibodies treatment (1 μg/ml). Scale bar; 200 μm (PDX_PC 110621). Scale bar; 50 μm (BxPC-3 and PDAC PKCY). D. Migration assay of PDX_PC 110621 cells with #132 (0.25 – 1 μg/ml). 20X magnification. E. Proliferation assay of PDX_PC 115026 (left graph) and PDX_PC 110621 (right graph) treated with anti-Gal-3BP antibodies (#13-7, #34, and #132) and control IgG (1 μg/ml). F. Proliferation assay of PDX_PC 115026+PSCs (left graph) and PDX_PC 110621+PSCs (right graph) treated with anti-Gal-3BP antibodies (#13-7, #34, and #132) and control IgG (1 μg/ml). * $p < 0.05$; ** $p < 0.01$; *** $p < 0.001$; ns (not significant)

Figure S2

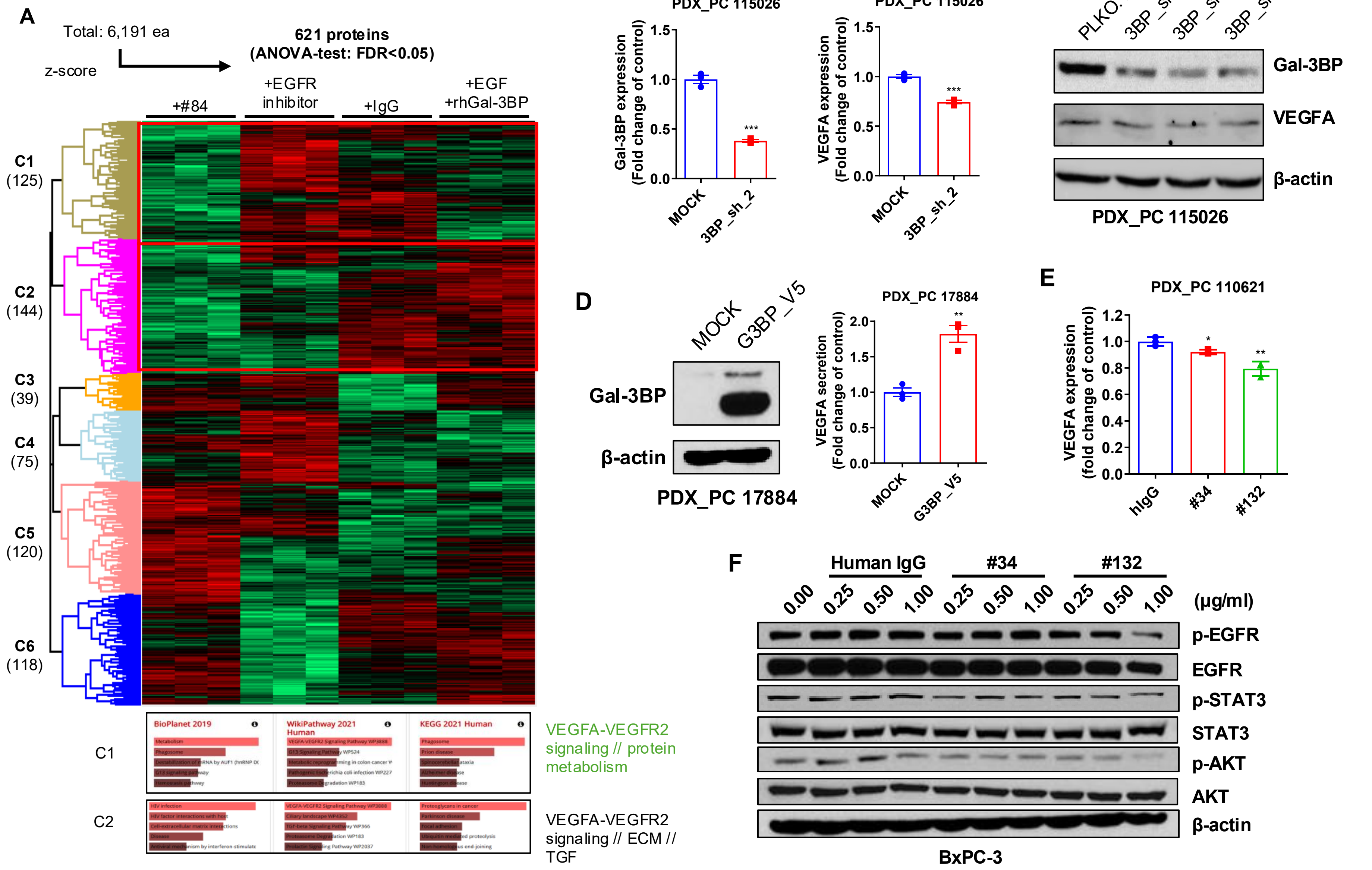

Supplementary Figure 2. Anti-Gal-3BP antibody inhibits VEGFA expression in PDAC cells.
A. Proteomic analysis for PDX_PC 110621 treated with #84, EGFR inhibitor, and control IgG presence rhGal-3BP+rhEGF. B,C. mRNA (B) and protein levels (C) of VEGFA in LGALS3BP knockdown PDX_PC 115026. D. secreted VEGFA levels in LGALS3BP-overexpressing PDX_PC 17884. E. VEGFA mRNA expression of PDX_PC 110621 treated with anti-Gal-3BP antibodies (#34 and #132) (1 μg/ml) F. Western blot for anti-Gal-3BP antibodies (#34 and #132) (0.25 - 1 μg/ml) treated BxPC-3 cells. * $p < 0.05$; ** $p < 0.01$; *** $p < 0.001$.

Figure S3

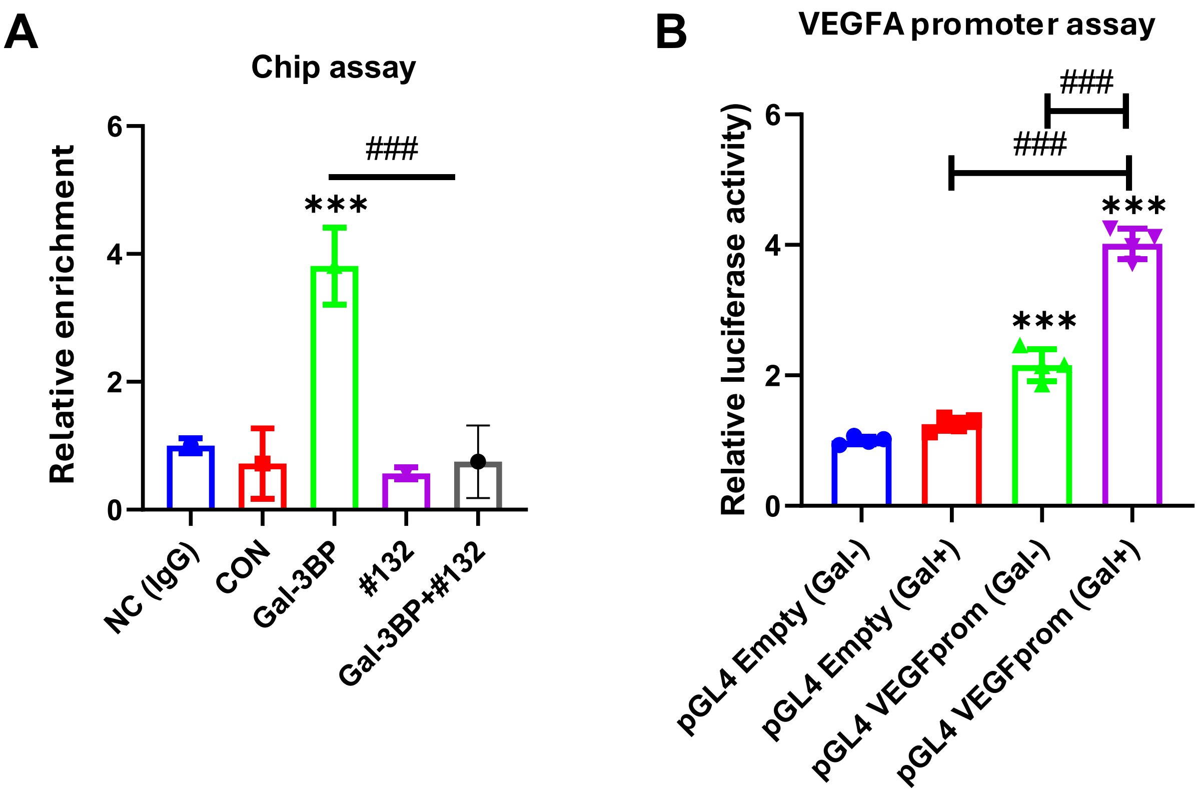

Supplementary Figure 3. Gal-3BP activates VEGFA promoter in PDAC cells.

A. Real time PCR analysis of ChIP assay in PancI treated with Gal-3BP, #132 and control IgG. B. Luciferase assay in PancI treated with Gal-3BP (Gal+) * $p < 0.05$; ** $p < 0.01$; *** $p < 0.001$ (compared to control). ### $p<0.001$ (paired analysis).

Figure S4

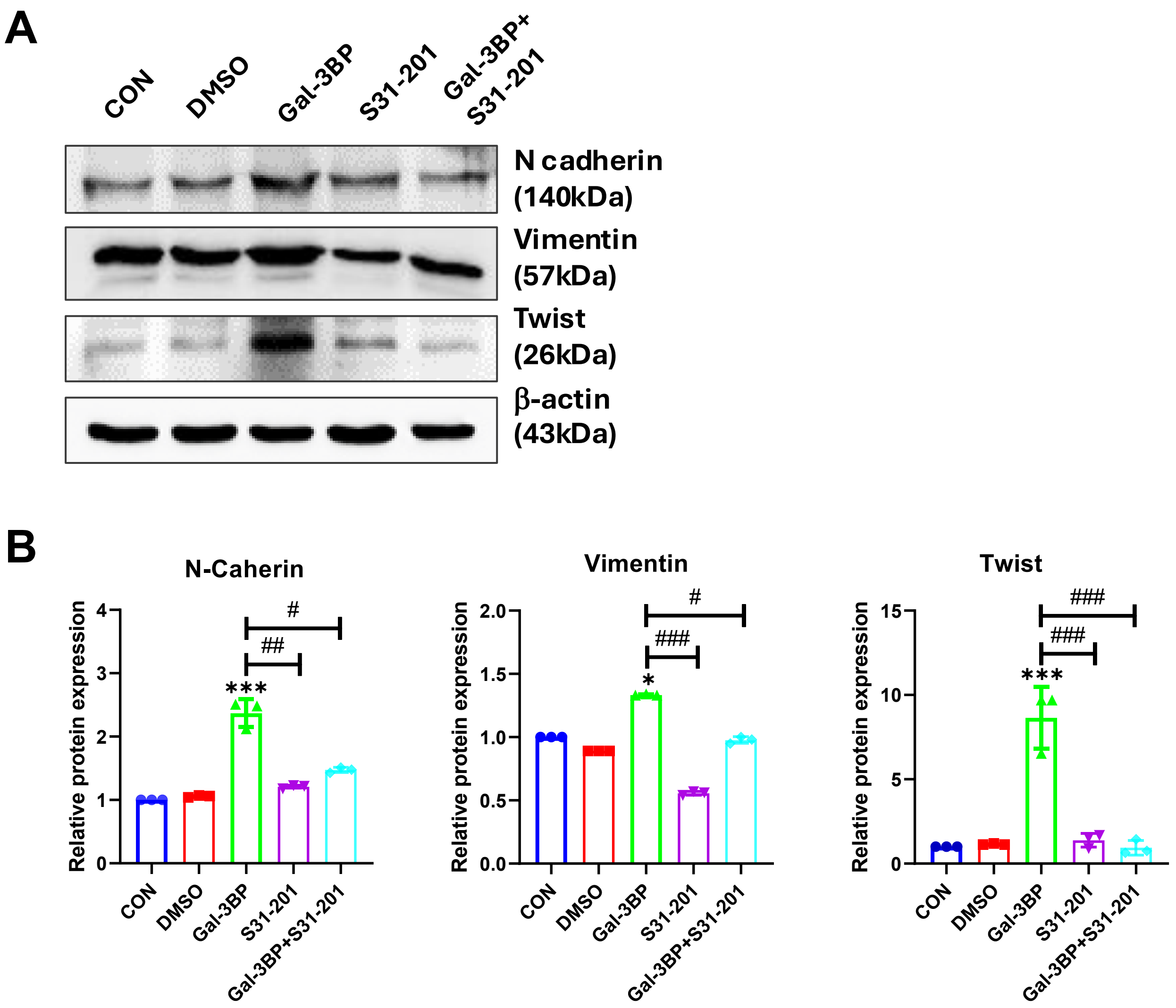

Figure S4. Gal-3BP/STAT3 signaling regulates EMT marker expression in PDAC cells.
A. WB analysis of epithelial–mesenchymal transition (EMT) markers in pancreatic ductal adenocarcinoma (PDAC) cells treated with Gal-3BP in the presence or absence of a STAT3 inhibitor (S31-201). B. Densitometric analysis of EMT marker expression was performed using ImageJ software. Band intensities were normalized to β-actin and are presented as fold change relative to the control group (mean ± SD, n = 3). #; p<0.05, ##; p<0.01, ###; p<0.001

Figure S5

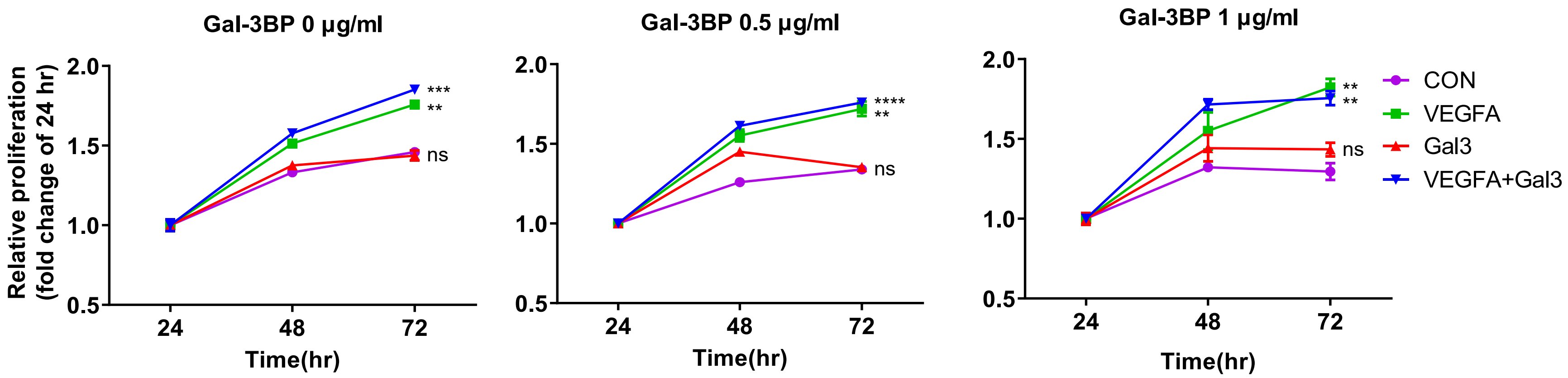

Supplementary Figure 5. Effec of Gal-3BP on HUVEC proliferation
The growth of HUVEC was measured after stimulation with VEGFA (Positive control, 50 ng/ml), rhGal-3 (1 μg/ml), and rhGal-3BP (1 μg/ml). The growth was monitored till 72hrs, in 24hr interval. ns. Not Significant ; ** $p < 0.01$; *** $p < 0.001$

Figure S6

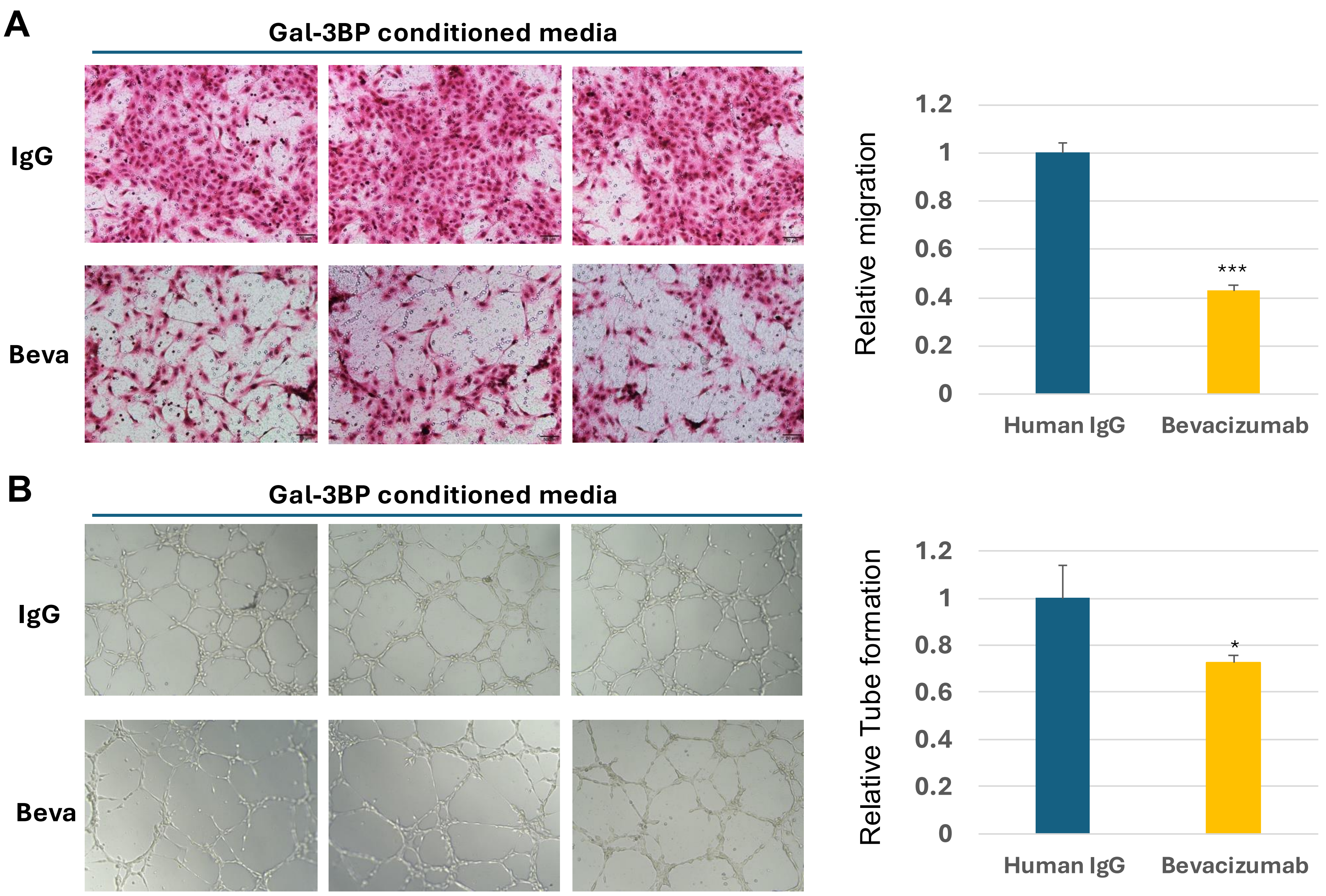

Supplementary Figure 6, Bevacizumab inhibits Gal-3BP induced HUVEC migration or tube formation
A, B. The migration (A) or tube formation (B) of HUVEC was measured after stimulation with Gal-3BP conditioned media with IgG or Bevacizumab (Beva). Left panel shows representative pictures. Graphs on right shows quantitation. * $p < 0.05$; *** $p < 0.001$

Figure S7

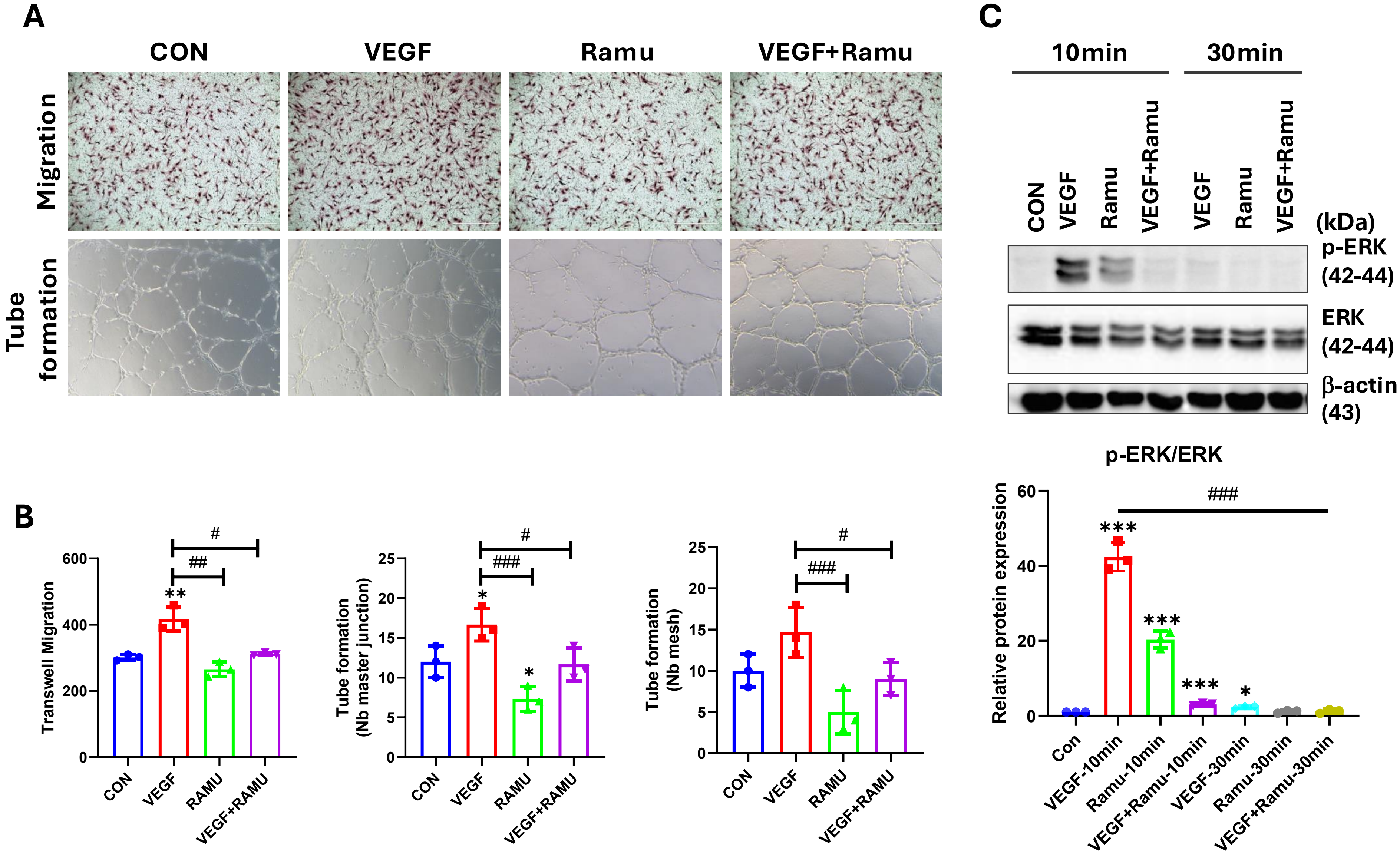

Supplementary Figure 7, Ramucirumab (VEGFR2 Ab) inhibits VEGFA-induced HUVEC migration or tube formation amd downstream p-ERK activation A. Representative pictures of migration (upper) or tube formation (lower) of HUVEC. B. Graphs for the measurement of image A. C. western blot of ERK and p-ERK for the HUVEC shown in panel A. Graph below the Wb panel shows densitometry quantitation. * $p < 0.05$; ** $p < 0.01$; *** $p < 0.001$ (to control) . $^{\#}$ $p < 0.05$; $^{\#\#}$$p < 0.05$; $^{\#\#\#}$ $p < 0.001$ (pairwise)

Figure S8

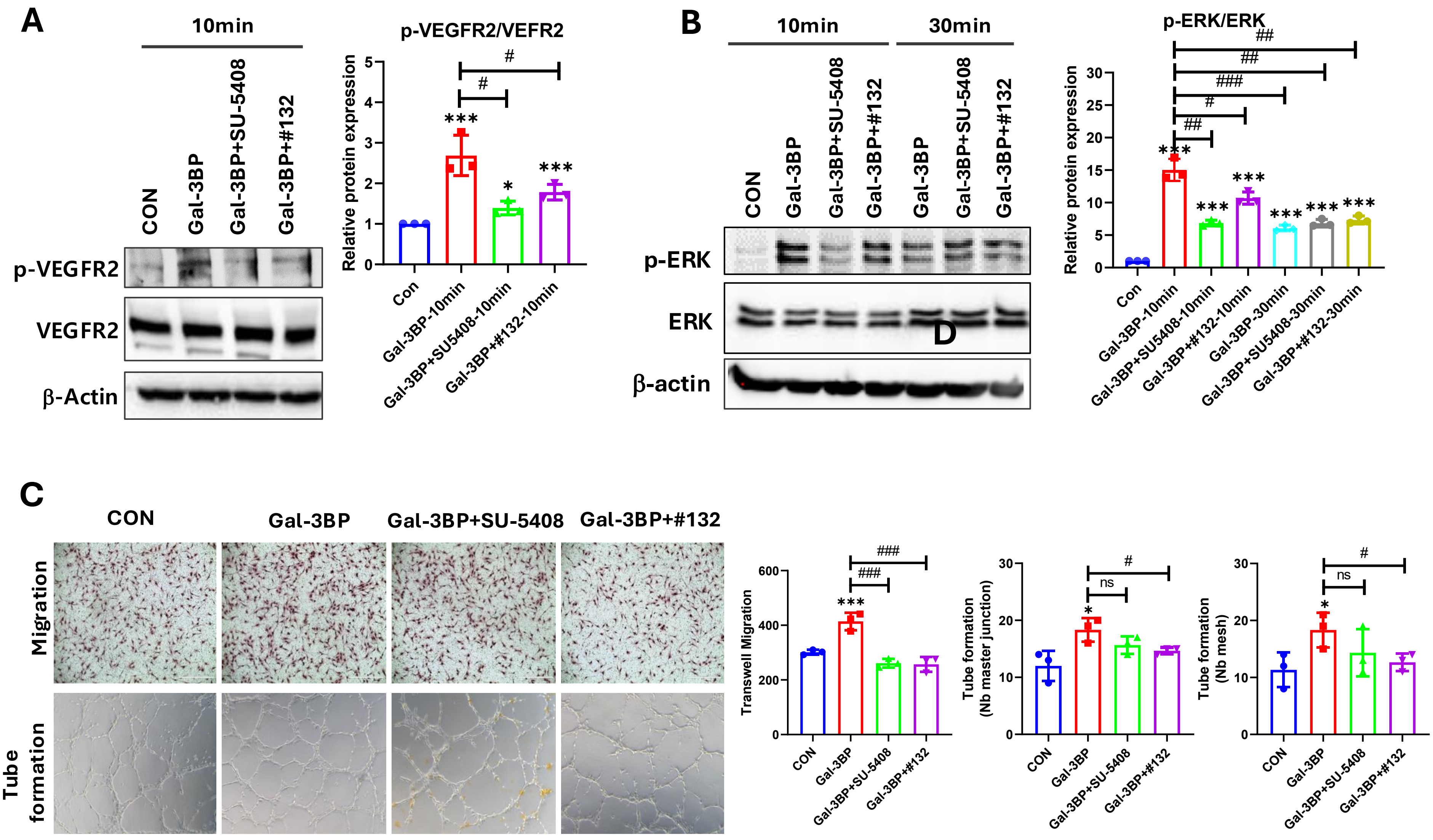

Supplementary Figure 8. VEGFR2 inhibitor SU-5408 inhibits Gal-3BP-induced HUVEC migration or tube formation along with downstream p-ERK suppression A, B. Western blot of p-VEGFR2 (A) or p-ERK (B) after Gal-3BP treatment in combination with SU-5408 or #132 Ab. Graphs on right show densitometry quantitation. C. Representative pictures of migration (upper) or tube formation (lower) of HUVEC. after the same treatment in A. Graphs on right show quantitation of the images. * $p < 0.05$; ** $p < 0.01$; *** $p < 0.001$ (to control) . # $p < 0.05$; ##$p < 0.05$; ### $p < 0.001$ (pairwise)

Figure S9

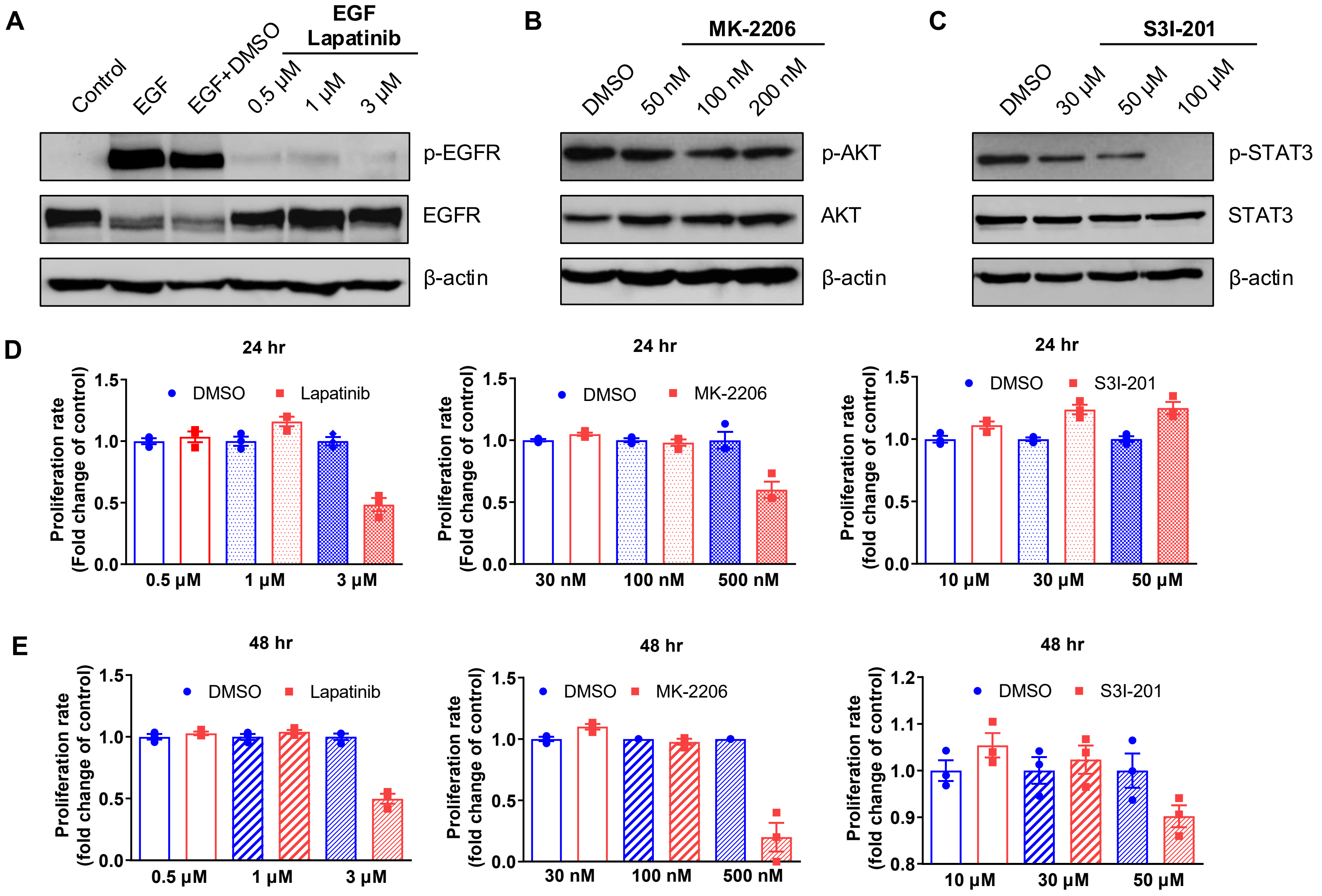

Supplementary Figure 9. phosphorylation and proliferation inhibitory effect of Lapatinib, MK-2206, and S3I-201
A. Protein expression of EGF (50 ng/ml) stimulated HUVEC with treatment of Lapatinib (0.5 μM, 1 μM, and 3 μM). B. Protein expression of MK-2206 (20 nM, 100 nM, and 200 nM) treated HUVEC. C. Protein levels of S3I-201 (30 μM, 50 μM, and 100 μM) treated HUVEC. D – E. HUVEC proliferation assay with treatment of Lapatinib (0.5 μM, 1 μM, and 3 μM), MK-2206 (30 nM, 100 nM, and 500nM), and S3I-201 (10 μM, 30 μM, and 50 μM) for 24 hr (D) and 48 hr (E).

Figure S10

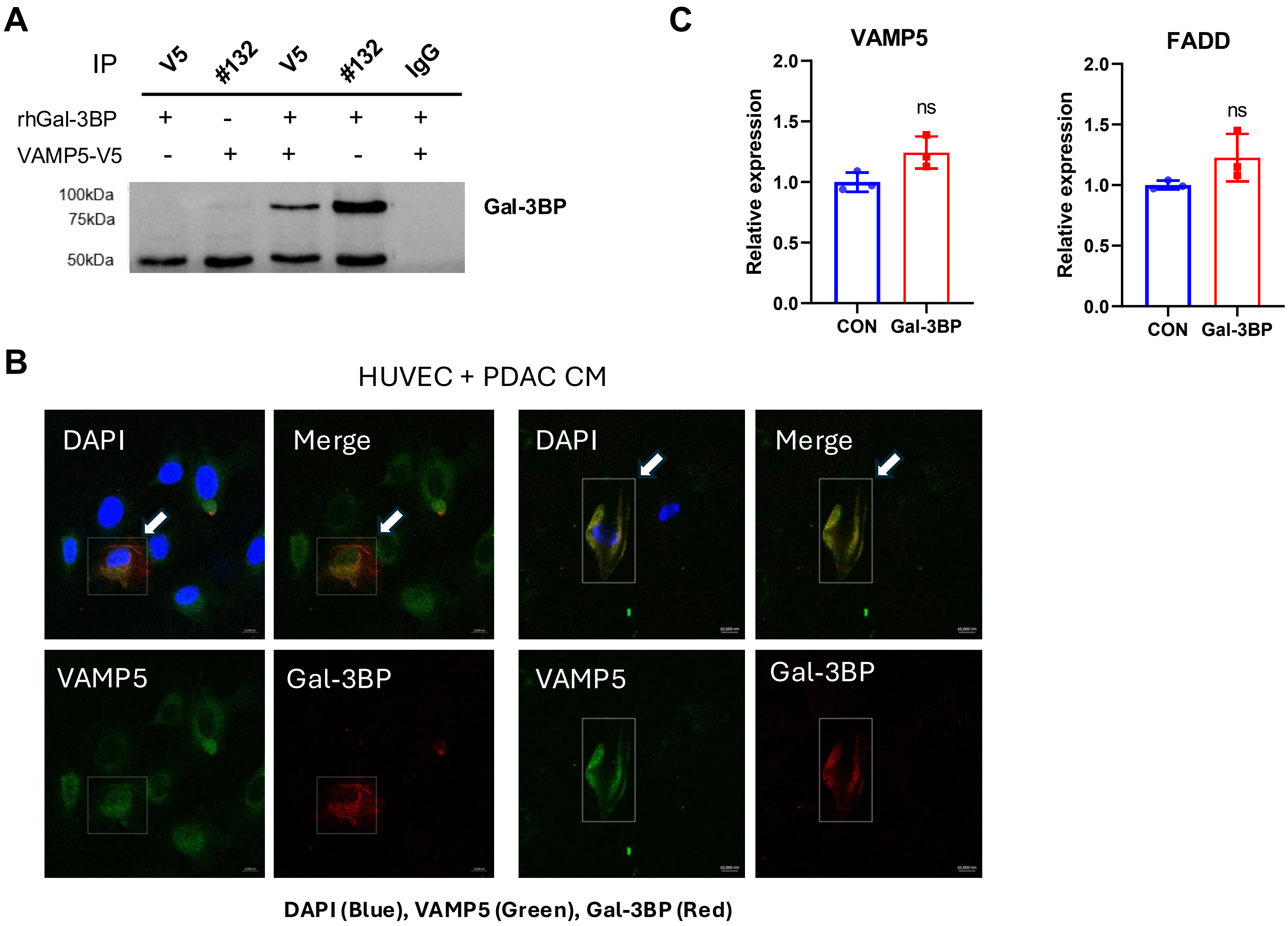

Supplementary Figure 10. Gal-3BP interacts with VAMP5. A. Western blot of Gal-3BP showing Co-IP between VAMP-5 and Gal-3BP. HEK 293 cells were overexpressed by VAPM5-V5 with the treatment of recombinant Gal-3BP. B. Immunocytochemistry in HUVEC for the colocalization study of Gal-3BP and VAMP5. DAPI (Blue), VAMP5 (Green), Gal-3BP #132 (Red). The arrow indicates cell with colocalized Gal-3BP and VAMP5, with yellow dots. C. real time PCR results for VAMP5 or FADD upon Gal-3BP treatment

Figure S11

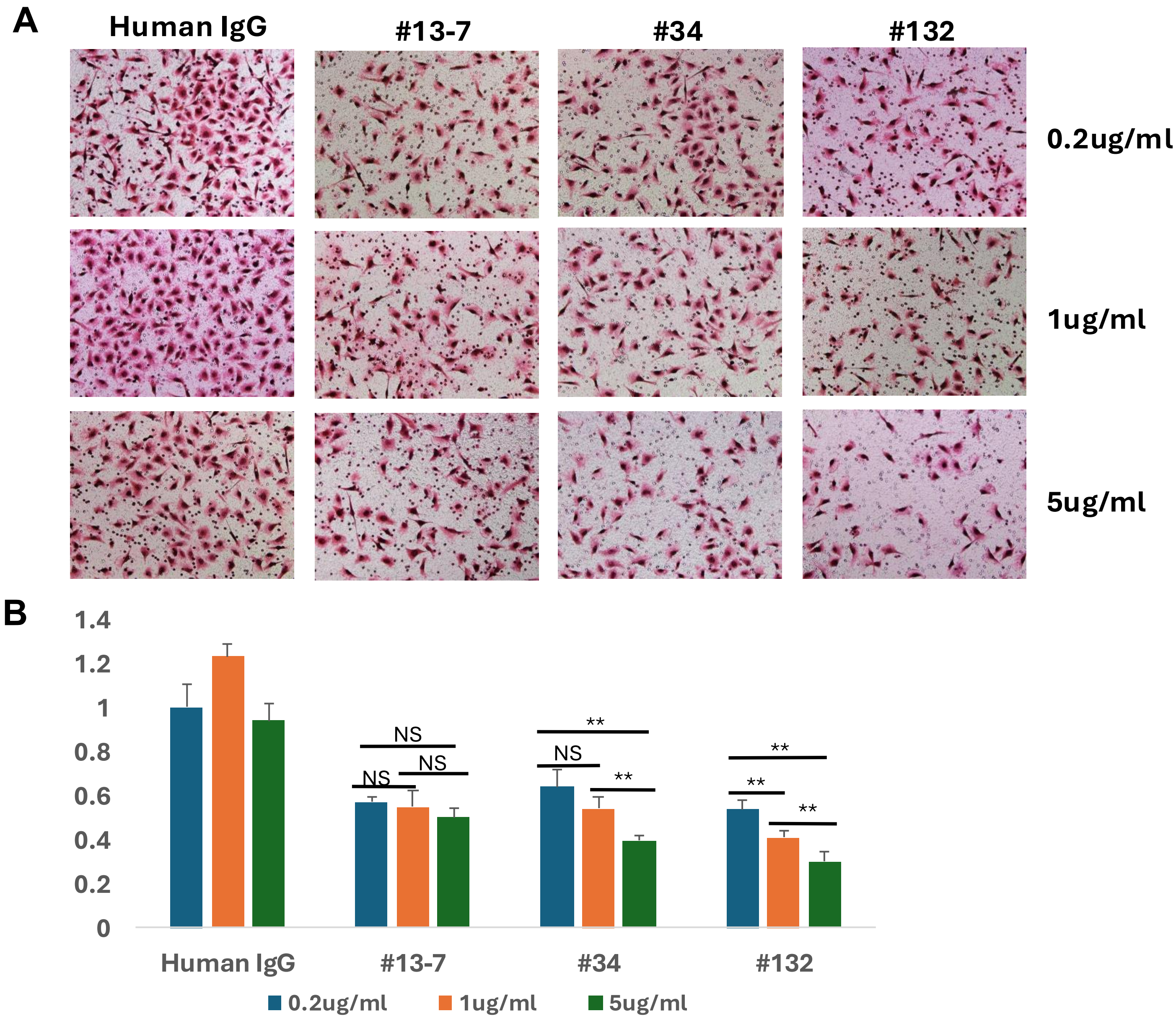

Supplementary Figure 11. Dose dependent inhibition of HUVEC migration by anti-Gal-3BP antibodies
A, Representative images of HUVEC migration by anti-Gal-3BP antibodies raging from 0.2 to 5ug/ml. B. Graph showing the quantitation of images in A. ** $p < 0.01$; ns not significant

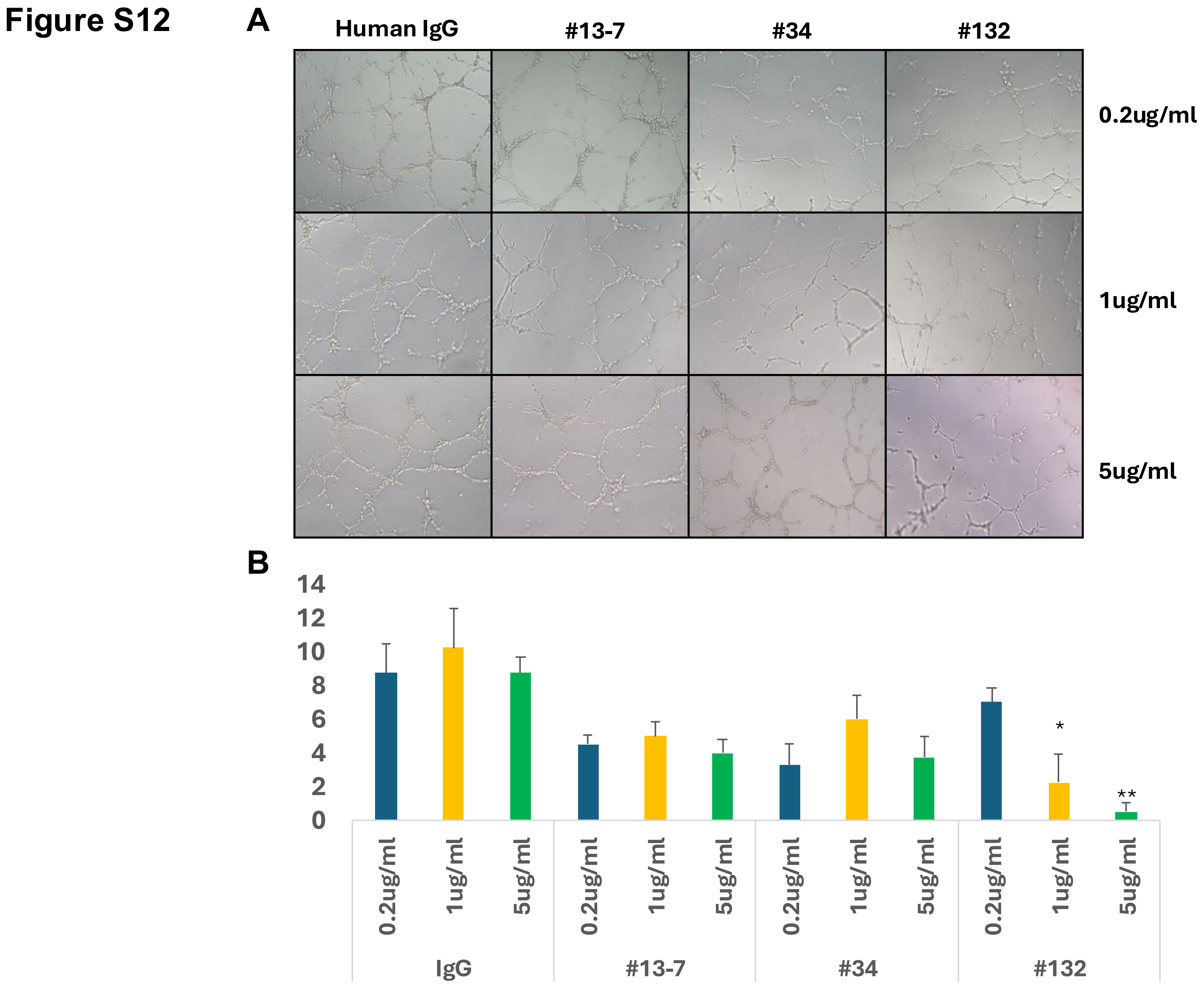

Supplementary Figure 12. Dose dependent inhibition of HUVEC tube formation by anti-Gal-3BP antibodies
A, Representative images of HUVEC tube formation by anti-Gal-3BP antibodies raging from 0.2 to 5ug/ml. B. Graph showing the quantitation of images in A. * $p < 0.05$; ** $p < 0.01$

Figure S13

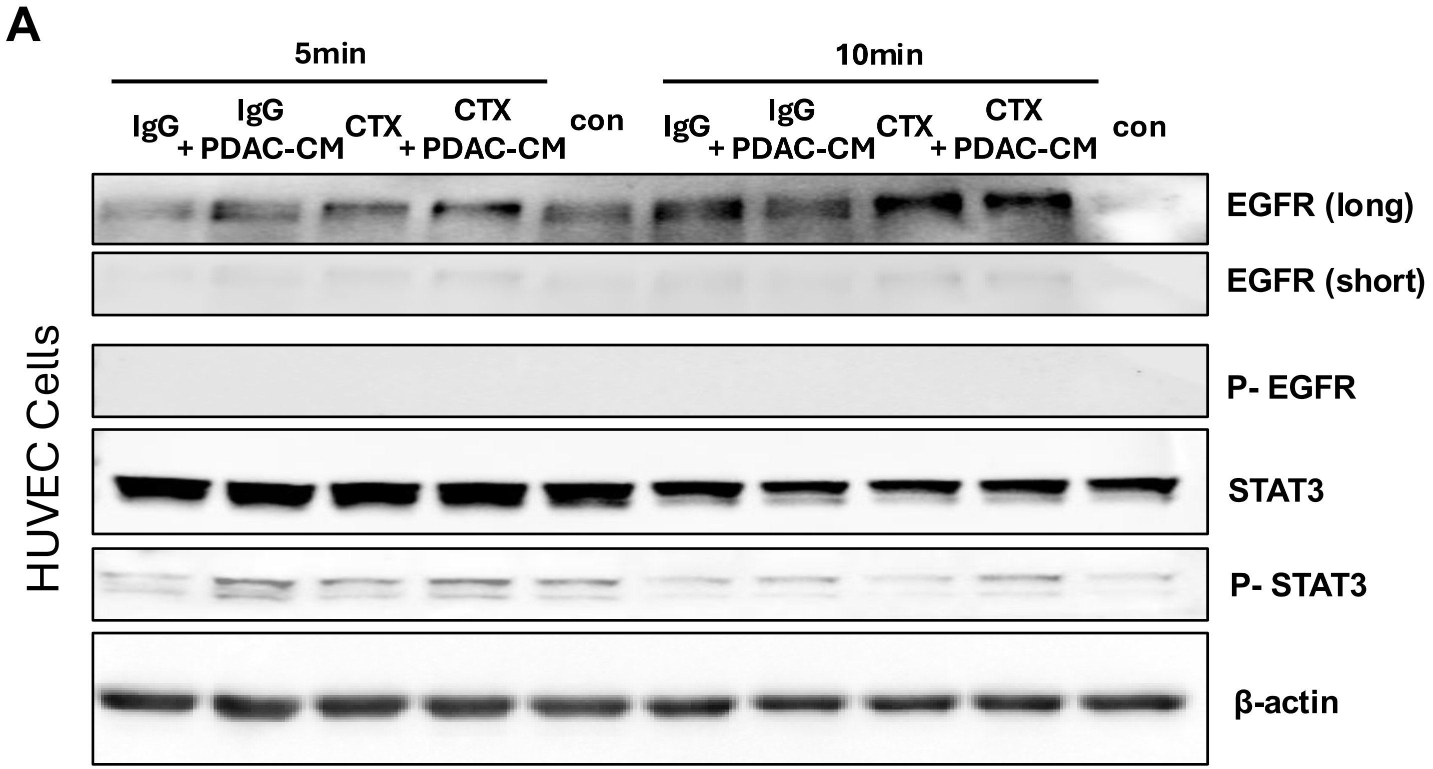

Supplementary Figure 13. HUVEC activation by Gal-3BP is EGFR independent. A. Western blot of EGFR, p-EGFR, STAT3 and p-STAT3 in HUVEC after treatment of Gal-3BP conditioned media in combination with EGFR inhibitor Cetuximab (CTX) or IgG. The analysis after 5,-min or 10min consistently show there is undetectable level of p-EGFR in HUVEC and CTX does not affect the p-STAT 3 level induced by Gal-3BP conditioned media.
